# Co-Treatment with Phlorotannin and Extracellular Vesicles from *Ecklonia cava* Inhibits UV-Induced Melanogenesis

**DOI:** 10.3390/antiox13040408

**Published:** 2024-03-28

**Authors:** Kyung-A Byun, Youngjin Park, Seyeon Oh, Sosorburam Batsukh, Kuk Hui Son, Kyunghee Byun

**Affiliations:** 1 Department of Anatomy & Cell Biology, College of Medicine, Gachon University, Incheon 21936, Republic of Korea; 2 LIBON Inc., Incheon 22006, Republic of Korea; 3 Functional Cellular Networks Laboratory, Lee Gil Ya Cancer and Diabetes Institute, Gachon University, Incheon 21999, Republic of Korea; 4 OBLIV CLINIC, Incheon 21998, Republic of Korea; 5 Department of Thoracic and Cardiovascular Surgery, Gachon University Gil Medical Center, Gachon University, Incheon 21565, Republic of Korea; 6 Department of Health Sciences and Technology, Gachon Advanced Institute for Health & Sciences and Technology (GAIHST), Gachon University, Incheon 21999, Republic of Korea

**Keywords:** extracellular vesicles from *Ecklonia cava*, melanogenesis, phlorotannin, TXNIP/NLRP3/IL-18 pathway, ultraviolet

## Abstract

Hyperpigmentation due to ultraviolet (UV)-induced melanogenesis causes various esthetic problems. Phlorotannin (PT) and extracellular vesicles (EVs) derived from various plants suppress melanogenesis pathways. We used UV-exposed keratinocytes and animal skin to determine if co-treatment with PT and EVs from *Ecklonia cava* (EVE) could inhibit melanogenesis by reducing UV-induced oxidative stress and the expression of the thioredoxin-interacting protein (TXNIP)/nucleotide-binding oligomerization domain-like receptor family pyrin domain containing the 3 (NLRP3)/interleukin-18 (IL-18) pathway, which are upstream signals of the microphthalmia-associated transcription factor. UV exposure increased oxidative stress in keratinocytes and animal skin, as evaluated by 8-OHdG expression, and this effect was reduced by co-treatment with PT and EVE. UV also increased binding between NLRP3 and TXNIP, which increased NLRP3 inflammasome activation and IL-18 secretion, and this effect was reduced by co-treatment with PT and EVE in keratinocytes and animal skin. In melanocytes, conditioned media (CM) from UV-exposed keratinocytes increased the expression of melanogenesis-related pathways; however, these effects were reduced with CM from UV-exposed keratinocytes treated with PT and EVE. Similarly, PT and EVE treatment reduced melanogenesis-related signals, melanin content, and increased basement membrane (BM) components in UV-exposed animal skin. Thus, co-treatment with PT and EVE reduced melanogenesis and restored the BM structure by reducing oxidative stress and TXNIP/NLRP3/IL-18 pathway expression.

## 1. Introduction

Melanin generated by melanocytes protects the skin from damage caused by ultraviolet (UV) radiation [1]; however, excessive melanin generation results in unwanted cosmetic effects such as dots, freckles, post-inflammatory hyperpigmentation, and senile lentigines [2]. Melanogenesis is a complex process involving various factors, including the microphthalmia-associated transcription factor (MITF), tyrosinase (TYR), tyrosinase-related protein 1 (TRP1), and tyrosinase-related protein 2 (TRP2) [3,4,5]. Skin inflammation and UV exposure also affect melanogenesis. For example, various inflammatory factors, such as interleukin (IL)-18 and IL-33, are known to increase melanogenesis [6,7]. IL-18 secreted from dendritic cells, macrophages, and keratinocytes [8,9,10] leads to the upregulated expression of protein kinase A (PKA) and p38/MAPK [8,11]. PKA and p38, in turn, activate MITF, an upstream controller of TRP1 and TRP2, which leads to increased melanogenesis in melanocytes [8,11].

One of the main factors that increase IL-18 synthesis is the inflammasome, which is a protein oligomer that propagates inflammatory reactions [12]. Nucleotide-binding oligomerization domain-like receptor family pyrin domain containing 3 (NLRP3), the most well-known inflammasome component, is a complex of the nucleotide-binding oligomerization domain-like receptor (NLR), apoptosis-associated speck-like protein containing a caspase recruitment domain (ASC), and pro-caspase 1 [13]. When an inflammasome is generated, inactive pro-caspase 1 turns into active caspase 1, which eventually produces active forms of IL-1β and IL-18 [14]. One type of stress that stimulates NLRP3 inflammasome formation is oxidative stress [15].

The main producer of reactive oxygen species (ROS) that stimulate NLRP3 inflammasomes is the nicotinamide-adenine dinucleotide phosphate (NADPH) oxidase system [16]. ROS also increases NLRP3 inflammasome activity by altering interactions between the thioredoxin-interacting protein (TXNIP) and thioredoxin (TRX) [17,18,19]. TRX is an endogenous antioxidant that is inhibited by binding with TXNIP [20,21]. Thus, TXNIP acts as a pro-oxidant that can induce cellular injury [22]. TXNIP activates NRLP3 inflammasomes by binding to the leucine-rich repeat domain of NLRP3 [23]. Oxidative stress promotes the dissociation of the binding between TXNIP and TRX, which eventually leads to NRLP3 inflammasome activation [23]. Conversely, the inhibition of TXNIP reduces NLRP3 inflammasome activation and inflammatory reaction [24]. Nuclear factor erythroid-2-related factor 2 (NRF2) helps control oxidative stress by upregulating antioxidant enzymes such as heme oxygenase-1 (HO-1) [25,26], which reduces ROS levels and binding between TXNIP and NLRP3 [27]. NRF2 also reduces inflammation by inhibiting NLRP3 inflammasome activation [28,29].

Chronic exposure to UV radiation induces melanogenesis by increasing oxidative stress and NLRP3 inflammasome activation in the skin [30]. In areas of skin with photoaged hyperpigmentation, the expression of NLRP3 and IL-18 is increased [31]. IL-18 is also involved in the upregulation of nuclear factor kappa B (NF-κB), which increases the expression of inflammatory factors such as interferon (IFN)-γ, IL-8, IL-1β, and tumor necrosis factor (TNF)-α [32,33,34,35,36]. The upregulation of NF-κB due to UV exposure leads to the upregulation of various matrix metalloproteinases (MMPs), which can destroy extracellular matrix (ECM) proteins such as collagen, elastin, and proteoglycans [37,38,39]. MMP2 and MMP9 are gelatinase proteins that destroy collagen type IV, which is a basement membrane (BM) component [40,41,42]. The destruction of the BM structure allows melanocyte migration into the dermis, which can induce persistent hyperpigmentation and various hyperpigmentation-related diseases such as melasma [43]. Unlike epidermal pigmentation, dermal pigmentation is not responsive to local treatments such as bleaching creams or laser therapies [44].

Nearly all types of cells secrete extracellular vesicles (EVs) consisting of a membrane bilayer enclosing various lipids, proteins, carbohydrates, and nucleic acids [45]. EVs are considered potential treatments for various diseases, including skin problems. EVs derived from mesenchymal stem cells reduced skin aging and the expression of senescence markers (p16, p21, IL-6, and IL-1β) [46]. Various plant-derived EVs (pEVs) contain biomolecules with antioxidant, antitumor, and immunomodulatory effects [47]. For example, pEVs from grapefruit, aloe vera, garlic, lavender, and ginger were shown to inhibit NLRP3 inflammasome activation and IL-1β and IL-18 secretion [48]. Ginger-derived EV also increased the activation of NRF2, which is involved in cell survival [49,50]. 

The pEVs also attenuate melanogenesis. EVs derived *Dendropanax morbifera* leaves decreased melanogenesis, which is associated with decreased TYR activity in the melanoma cell [51]. EVs from ginseng roots decreased melanin amount in UV-radiated human epidermal melanocytes [52]. EVs derived from *Sargassum fusiforme* and *Codium fragile* reduced the expression of TRP1 and MITF in MNT-1 human melanoma cells [53].

Moreover, phlorotannin (PT), which refers to polyphenols found in brown algae, has an anti-pigmentation effect [54]. Brown alga-derived PT such as phloroglucinol, eckol, dieckol, diphlorethohydroxycarmalol, and octaphlorethol A are known to act as potent TYR inhibitors [55,56,57,58,59]. PT isolated from *Schizymenia dubyi*, *Endarachne binghamiae*, and *Sargassum siliquastrum* inhibits tyrosinase [60]. An extract from the brown seaweed *Ecklonia cava,* which contained PT, reduced melanogenesis by reducing MITF, TYR, TRP1, and TRP2 levels [61]. The extract from *E. cava* also increased NRF2 and HO-1 levels and reduced oxidative stress in myoblasts [62]. Moreover, PT from *Ecklonia stolonifera* reduced melanin synthesis by reducing TRP1 and TRP2 levels in B16F10 melanoma cells [63].

Because the extract from *E. cava* is known to increase NRF2, and various pEVs are known to reduce NLRP3 inflammasome formation, we hypothesized that EVs from *E. cava* (EVE) can increase NRF2 levels and reduce oxidative stress, leading to downstream reductions in NLRP3 inflammasome activity, IL-18 secretion, PKA and p38 signaling, MITF activation, TYR, TRP1, and TRP2 levels, and, ultimately, melanogenesis, BM destruction, and dermal pigmentation. To test this hypothesis, we evaluated the effect of EVE on skin pigmentation using an in vitro model of UV-exposed keratinocytes and melanocytes and an in vivo model of UV-exposed animal skin. We also tested whether a combination treatment with PT and EVE could reduce melanogenesis more than treatment with EVE or PT alone.

## 2. Materials and Methods

### 2.1. PT Preparation

PT was obtained from the *E. cava* extract provided by Aqua Green Technology Co., Ltd. (Jeju, Republic of Korea) as in previous research [64]. The *E. cava* extract was prepared as follows. *E. cava* was washed and air dried at room temperature for 48 h. Extraction was performed at 85 °C for 12 h using a 50% ethanol solvent. The extract was then filtered and concentrated [65].

### 2.2. EVE Preparation and Analysis

#### 2.2.1. EVE Preparation

EVE was obtained from *E. cava* material provided by Aqua Green Technology Co., Ltd. The *E. cava* material was dissolved in distilled water (DW) at a ratio of 1:30, and extraction was performed by stirring at 50 °C for 24 h. The extract was centrifuged at 3000× *g* for 30 min at room temperature. The supernatant was transferred to an ultra-clear tube (Beckman, Brea, CA, USA), and vacuoles were removed by high-speed centrifugation at 50,000× *g* for 90 min. The supernatant from which the vacuoles were removed was then placed in a new ultra-clear tube and ultracentrifuged at 10,000× *g* for 120 min to obtain pellet-containing exosomes. Finally, the pellet was diluted in DW and ultracentrifuged at 10,000× *g* for 120 min to obtain a clean pellet of EVs.

#### 2.2.2. Nanoparticle-Tracking Analysis

To measure the number of particles, EVE was prepared by serial dilutions 20×, 200×, and 2000× with DW. While removing air bubbles in the syringe, the solution was injected into the equipment (NanoSight NS300; Malvern Panalytic LTD., Malvern, UK) by slowly pressing the syringe. The concentration range of the equipment was 10^5^–10^9^ particles/mL. The data are presented in Table S1.

### 2.3. In Vitro Experiments

#### 2.3.1. Cell Culture

HaCaT keratinocytes were distributed and used by Professor Jeong Hee Hong’s team at Gachon University. The keratinocytes were cultured in Dulbecco’s modified Eagle’s medium (DMEM; HyClone, Logan, UT, USA) at 37 °C with 5% CO_2_. Primary human epidermal melanocytes (HEMn) were purchased from American Type Culture Collection (ATCC; Manassas, VA, USA) and cultured in a Dermal Cell Basal Medium with a melanocyte growth kit (ATCC) at 37 °C with 5% CO_2_.

#### 2.3.2. Experimental Design

In vitro experiments were conducted in four stages to test the effects of PT and EVE on keratinocytes and melanocytes. First, to determine the optimal concentrations of PT and EVE, a keratinocyte model was designed in which keratinocytes were exposed to UV radiation for 30 s (UV lamp with 306 nm peak wavelength; Sankyo, Yokohama, Japan) and then incubated for 48 h with phosphate-buffered saline (PBS) or 25, 50, or 100 μg/mL of PT or 7.5 × 10^7^, 1.5 × 10^8^, or 3.0 × 10^8^ particles/mL of EVE, and cell lysates were obtained for mRNA analysis (Figure S1). Second, for melanocyte proliferation experiments, melanocytes were treated with α-MSH (200 nM; Sigma-Aldrich, St. Louis, MO, USA) for 24 h and then incubated for 48 h with PBS or 25, 50, or 100 μg/mL of PT or 7.5 × 10^7^, 1.5 × 10^8^, or 3.0 × 10^8^ particles/mL of EVE (Figure S1). 

Third, after the optimal concentrations of PT and EVE were confirmed (50 μg/mL, 1.5 × 10^8^ particles/mL in each case; Figure S2), keratinocytes were exposed to UV radiation and then incubated for 48 h with PBS, 50 μg/mL of PT, 1.5 × 10^8^ particles/mL of EVE, or 50 μg/mL of PT and 1.5 × 10^8^ particles/mL of EVE. Control groups that were not exposed to UV radiation were incubated for 48 h with PBS. After the 48 h incubation, cell lysates were collected for protein or RNA analysis, and supernatants were collected to use as conditioned media (CM) for melanocytes. Fourth, a melanocyte model was designed in which melanocytes were treated for 48 h with CM from each of the five treatment groups in the keratinocyte model (Figure S3). Cell lysates were then collected from all melanocyte groups for protein or RNA analysis.

### 2.4. In Vivo Experiments

#### 2.4.1. Mouse Model and Maintenance

Female HRM-2 (6 weeks old) mice were obtained from the Central Laboratory Animal Center (Incheon, Republic of Korea) and stabilized in our facility for 2 weeks before the experiments. All animals were housed under conditions of constant temperature 20–24 °C and humidity 45–55% and were allowed to consume food and water freely. This study was conducted with approval from the Gachon University Animal Experiment Ethics Committee (IACUC, approval number LCDI-2023-0050).

#### 2.4.2. Experimental Design

Stabilized animals were randomly divided into five groups. Four groups were exposed to UV radiation, as described in a previous study [66]. Briefly, a UV lamp (Sankyo) with a 306 nm peak wavelength was used to apply 200 mJ/cm^2^ of UV radiation to the backs of the mice once every two days for 10 days and then every day for the next three days. Then, DW, 1 mg/mL of PT, 3.0 × 10^10^ particles/mL of EVE, or 1 mg/mL of PT and 3.0 × 10^10^ particles/mL of EVE were administered once to a 2 cm × 2 cm patch on the backs of mice using a microneedling system (MTS), and the mice continued to receive UV radiation every other day. The total injection volume of all solutions was 200 μL. After 28 days, the animal skin was harvested (Figure S4).

#### 2.4.3. Skin Lightness

Skin color was measured using a CR-10 color reader (Konica Minolta Sensing, Inc., Sakai, Osaka, Japan), and L* (i.e., brightness) was measured in the CIELAB color space (International Commission on Lighting, Vienna, Austria). The measurement cycle was averaged by measuring 10 times on the 42nd day (28 days after the start of topical treatment) after UV radiation was first applied.

### 2.5. Sample Preparation

#### 2.5.1. Transmission Electron Microscopy (TEM)

For EVE, a drop of EVE diluted with DW was placed on a Formvar carbon-coated grid for 15 s, and moisture was removed using filter paper. Then, one drop of 1% urinyl acetate was added for 15 s, and moisture was removed using filter paper. Finally, the grid was washed with a drop of DW.

For skin, the tissue was cut into 1 mm × 1 mm pieces and fixed in 2% glutaraldehyde/2% paraformaldehyde in a 0.1 M phosphate buffer (pH 7.4) for 24 h. After washing with the 0.1 M phosphate buffer, the skin sections were fixed in 1% OsO_4_ in a 0.1 M phosphate buffer for 2 h and dehydrated in an ethanol series (50, 60, 70, 80, 90, 95, 100%; 10 min each). The sections were then permeated with propylene oxide for 10 min, embedded with the poly/bed 812 kit (Polysciences, Inc., Warrington, PA, USA) for 12 h, and polymerized in an electron microscope oven at 65 °C for 12 h. The block was sectioned into 200 nm sections using a diamond knife on an ultramicrotome and stained with toluidine blue for light microscopy. The sections were thinned to 80 nm using an ultramicrotome, placed on a copper grid, stained with 3% uranyl acetate for 30 min, and double stained with 3% lead citrate for 7 min.

#### 2.5.2. RNA Extraction

RNA was extracted from cells and skin tissues according to the instructions of the RNAiso reagent manufacturer (TAKARA, Tokyo, Japan). Briefly, for cells, samples were washed three times with PBS and then homogenized at room temperature for 5 min by adding 1 mL of RNAiso. For skin, 40 mg of tissue was cut into several pieces, diluted with 1 mL of RNAiso, and homogenized by sonication with 10 cycles of 40 s/60 s working/resting time. All samples were then centrifuged at 12,000× *g* at 4 °C for 5 min, and the supernatant was transferred to a new tube. After vortexing with 0.2 mL of chloroform (Samchun, Pyeongtaek, Republic of Korea), the tube was incubated at room temperature for 5 min and centrifuged at 4 °C and 12,000× *g* for 15 min. The supernatant was transferred to a new tube, and 0.5 mL of isopropanol (Duksan, Seoul, Republic of Korea) was added. The tube was shaken, incubated at room temperature for 10 min, and centrifuged at 4 °C and 12,000× *g* for 10 min. The supernatant was removed, 1 mL of 75% ethanol (Supelco, St. Louis, MO, USA) was added to the remaining pellet, the tube was shaken several times, and the pellet was washed by centrifugation at 7500× *g* for 5 min at 4 °C. The liquid was then thoroughly removed, the remaining pellet was air-dried, and an appropriate amount of diethylpyrocarbonate-treated water (Biosesang, Seongnam, Republic of Korea) was added.

#### 2.5.3. cDNA Synthesis

cDNA synthesis was carried out according to the instructions of the cDNA synthesis kit (TAKARA). First, the concentration and purity of RNA were measured using a Nanodrop spectrophotometer (Thermo Fisher Scientific, Waltham, MA, USA). Then, 1 μg of RNA was mixed with RNase-free distilled water, an oligo DT primer, and dNTP and incubated at 65 °C for 5 min. Reverse transcriptase and an RNase inhibitor were then added, and the mixture was incubated at 42 °C for 45 min, 95 °C for 5 min, and then cooled at 4 °C.

#### 2.5.4. Protein Isolation

Protein extraction was carried out according to the instructions of the EzRIPA buffer kit (ATTO Corporation, Tokyo, Japan). EVE was washed with PBS and diluted with a 1 mL RIPA buffer, and cells were washed with PBS and scraped with a 1 mL RIPA buffer. For the skin, 40 mg of tissue was cut into several pieces and diluted with a 1 mL RIPA buffer, homogenized with 10 cycles of sonication with 40 s/60 s working/resting time and then incubated on ice for 10 min to promote protein solubilization. Cell and tissue samples were then sonicated (high power, 10 s/60 s working/resting time) and centrifuged at 14,000× *g* for 15 min at 4 °C to isolate the proteins. Protein concentration was measured using a bicinchoninic acid assay kit (BCA kit; Thermo Fisher Scientific).

#### 2.5.5. Co-Immunoprecipitation (Co-IP)

Washed cells were scraped into an NP buffer (GenDEPOT, Katy, TX, USA) supplemented with protease and phosphatase inhibitors. Additionally, skin tissue (100 mg) was homogenized in the same NP buffer using a bead homogenizer. After this, the extraction process was the same as the protein extraction process. The protein concentration was measured using a BCA kit.

#### 2.5.6. Paraffin-Embedded Skin Tissue Block

Skin tissue was fixed in cold 4% paraformaldehyde (Sigma-Aldrich) for 48 h, placed in a cassette, and washed with DW. Then, the sample was placed in a tissue processor (Leica, Wetzlar, Germany), sequentially soaked in 95% and 99% ethanol (Duksan), dehydrated, dipped in xylene (Duksan), and infiltrated with paraffin (Leica). Tissue blocks soaked in paraffin were made into paraffin blocks in an embedding machine, sectioned to a thickness of 7 µm using a microtome (Leica), placed on a coated slide, incubated overnight at 60 °C, and attached to the slide.

### 2.6. TEM

TEM and Cryo-TEM imaging was performed as follows. For Cryo-TEM, a Leica EM ACE600 (Leica microsystem) was used for the glow discharge of the cryo-TEM grid (200 mesh CF-1.2/1.3 Au, EMS). Then, 5 μL of EVE was added onto the grid, and the sample was plunged frozen into liquid ethane after the excess fluid was removed by automatic blotting in a Leica EM GP2 (Leica microsystems). The grid was loaded into an Elsa cryo-transfer holder (Gatan, Inc., Pleasanton, CA, USA), and EVE sample was analyzed with an HT7800 cryo-TEM (HITACHI, Tokyo, Japan) at the Yonsei Biomedical Research Institute, Yonsei University College of Medicine. For TEM, the stained EVE and skin samples grids were imaged with JEM-1011 TEM (JEOL, Tokyo, Japan) [67].

Quantitative analysis on the amount of lamina densa destruction and melanin was performed using ImageJ software version 1.53s (National Institutes of Health; NIH, Bethesda, MD, USA). The amount of lamina densa destruction and melanin in several fields was measured and quantified. Each group was compared to a control sample.

### 2.7. Cell Proliferation and Cell Viability

To test the proliferation of PT and EVE, melanocytes were seeded in a 96-well plate (2 × 10^4^ cells/well). After 24 h, the cells were treated with α-MSH (200 nM) for 24 h and then treated for 48 h with PBS or 25, 50, or 100 μg/mL of PT or 7.5 × 10^7^, 1.5 × 10^8^, or 3.0 × 10^8^ particles/mL of EVE. The medium was then removed, and the cells were washed with Dulbecco’s phosphate-buffered saline (DPBS; Gibco, Waltham, MA, USA). Then, 10 µL of the cell counting kit-8 (CCK-8; Sigma-Aldrich) reagent and 90 µL of the growth medium were added to each well, and the cells were incubated at 37 °C for 1.5 h. Optical density was measured using a microplate reader at 450 nm. Each analysis was performed in triplicate.

To test the cytotoxicity of PT and EVE, keratinocytes were seeded in a 96-well plate (1 × 10^4^ cells/well). When the wells were 100% filled with cells, the cells were treated for 24 h with PT at concentrations of 1, 10, 100, 500, and 1000 μg/mL or EVE at concentrations of 3 × 10^7^, 3 × 10^8^, 3 × 10^9^, 1.5 × 10^10^, and 3.0 × 10^10^ particles/mL. The medium was then removed, and the cells were washed with DPBS (Gibco). Then, a 10 µL CCK-8 (Sigma-Aldrich) reagent and 90 µL growth medium were added to each well, and the cells were incubated at 37 °C for 2 h. Optical density was measured using a microplate reader at 450 nm. Each analysis was performed in triplicate.

### 2.8. Quantitative Reverse Transcription–Polymerase Chain Reaction (qRT-PCR)

For qRT-PCR, 2.5 µL of cDNA template, 5 µL of SYBR green premix (TAKARA), 0.4 µL each of reverse and forward primers (Table S2), and 1.7 µL of DW were mixed and dispensed into 384 wells (Thermo Fisher Scientific) so that the total volume in each well was 10 µL. The qRT-PCR process and melting curve analysis were performed using a QuantStudio^TM^ 3 real-time PCR instrument (Thermo Fisher Scientific). The qRT-PCR amplification procedure consisted of initial denaturation at 95 °C for 10 min, followed by 40 cycles of denaturation at 95 °C for 15 s, annealing at 60 °C for 1 min, and denaturation at 95 °C for 15 s. Afterwards, melt analysis was performed over a temperature range of 60 °C to 95 °C at an increment rate of 0.075 °C/s. Gene expression levels were quantified using the comparative cycle threshold (CT) method (ΔΔCT). mRNA levels were normalized to *ACTB*/*Actb* gene and compared with levels in the control.

### 2.9. Western Blot

Fifty micrograms of PT or EVE or 30 μg of cell lysate or skin protein were combined with a 4× LDS sample buffer (Thermo Fisher Scientific) and 10× sample-reducing agent (Thermo Fisher Scientific). The protein mixture was heated at 70 °C for 10 min, and the denatured proteins were subjected to 10% sodium dodecyl sulfate–polyacrylamide gel electrophoresis (SDS-PAGE) for 25 min at 200 V using the MOPS buffer (Invitrogen, Waltham, MA, USA). The separated proteins were transferred to a PVDF membrane (Millipore, Burlington, MA, USA) using a semi-dry transfer system at a current of 1 A for 10 min. To inhibit non-specific binding, the PVDF membrane was incubated with 5% Skim Milk (LPS Solution, Daejeon, Republic of Korea) in 0.1% Tween 20 (SPL, Pocheon, Republic of Korea) in tris-buffered saline (TTBS) at room temperature for 1–2 h. The membrane was washed three times with 0.1% TTBS and incubated with appropriately diluted primary antibodies overnight at 4 °C (Table S3). After three washes with 0.1% TTBS, the membrane was incubated with the horseradish peroxidase-conjugated secondary antibody (1:1000; Vector Laboratories, Newark, CA, USA) for 1 h at room temperature. Protein bands were visualized using chemiluminescent solutions and identified with a ChemiDoc Imaging System (Bio-Rad, Hercules, CA, USA). 

For the quantitative analysis of proteins, band intensity was quantified using ImageJ software version 1.53s (NIH). Beta-actin bands were used to demonstrate equivalent loading control. Each group was compared with the control sample [68].

### 2.10. Enzyme-Linked Immunosorbent Assay (ELISA)

Microplates were incubated overnight at 4 °C with 100 nM of carbonate and a bicarbonate-mixed buffer (pH 9.6) and washed three times with 0.1% Tween 20 in phosphate-buffered saline (TPBS) to remove the unattached material. To prevent non-specific protein binding, the microplates were incubated with 5% Skim Milk (LPS Solution) in 0.1% TPBS overnight at 4 °C. After washing three times with 0.1% TPBS, 30 μg of the protein sample was added to each well and incubated overnight at 4 °C. The wells were then washed with 0.1% TPBS and incubated overnight at 4 °C with primary antibodies diluted in PBS (Table S3). After washing with PBS, the horseradish peroxidase-conjugated secondary antibody (1:1000; Vector Laboratories) was added and incubated at room temperature for 4 h. To confirm its expression, tetramethylbenzidine (TMB) solution (Sigma-Aldrich) was applied to each well and incubated for 15–20 min at room temperature. To stop the reaction, a stop solution consisting of 2 N of sulfuric acid (Sigma-Aldrich) was used. Finally, measurements were made using a microplate reader at 450 nm. Each analysis was performed in triplicate.

### 2.11. Co-IP

Proteins were incubated with 10 μL A/G agarose beads (GenDEPOT) for 1 h with gentle rotation at 4 °C and centrifuged at 2500 rpm for 15 min at 4 °C to separate the beads and proteins with non-specific binding. The protein samples (1 mg) were then incubated with 1 μg of the TXNIP primary antibody overnight at 4 °C with gentle rotation. Protein A/G agarose beads were added to the mixture, and the mixture was incubated overnight at 4 °C to collect antibody–protein complexes. The mixture was then centrifuged at 2500 rpm for 15 min at 4 °C, the supernatant was discarded, and the pellet was washed with a cold NP buffer to remove non-specifically bound proteins. Then, a 4× LDS sample buffer (Invitrogen) and 10× sample-reducing agent (Invitrogen) were added to the beads, and the mixture was incubated at 70 °C for 10 min to extract the proteins. The samples were centrifuged at 2500 rpm for 15 min at 4 °C and evaluated by Western blot, as in Section 2.9 [69].

### 2.12. Staining

#### 2.12.1. Immunohistochemistry

Skin tissue sections were deparaffinized and rehydrated by sequential transfer to xylene and 100–70% ethanol. The sections were boiled in a sodium citrate buffer (pH 6.0; Sigma-Aldrich) in a microwave oven for 5 min and cooled in DW for antigen retrieval. After three PBS washes, non-specific binding was blocked by incubation with serum solution for 1 h at room temperature, and the slides were incubated with primary antibodies overnight at 4 °C (Table S2). After washing with PBS, the slides were incubated with biotinylated secondary antibodies (Vector Laboratories) for 1 h at room temperature. The slides were then rinsed with PBS, incubated with an ABC reagent (Vector Laboratories), washed, and incubated with a 3,3′-Diaminobenzidine solution (Sigma-Aldrich) for 5 min, resulting in a brown reaction. For counterstaining, the slides were incubated with hematoxylin (KPNT, Cheongju, Republic of Korea) for 30 s, washed with DW, dehydrated, and mounted using a DPX mounting solution (Sigma-Aldrich). Finally, the stained tissue was scanned using a slide scanner (Motic Scan Infinity 100; Motic, Beijing, China) and was randomly captured.

The quantitative analysis of proteins was performed using ImageJ software version 1.53s (NIH). The yellow-to-brown color was regarded as positive staining. The brown color was extracted from the image and converted to black to quantify the intensity of the image. Each group was compared with the control sample [70].

#### 2.12.2. Fontana Masson

Fontana Masson staining was performed according to the manufacturer’s instructions (Scytek, Logan, UT, USA). Briefly, skin tissue sections were deparaffinized and rehydrated by sequential transfer to xylene and 100–70% ethanol. Sections were then incubated in a Fontana ammonia silver solution for 30 min at 60 °C. Then, after rinsing three times with DW, non-melanin-stained areas were removed with a 0.2% gold chloride solution and 5% sodium thiosulfate solution. Nuclei were stained with Nuclear Fast Red solution, and the sections were dehydrated and mounted using the DPX mounting solution (Sigma-Aldrich). Finally, the stained tissue was scanned using a slide scanner (Motic Scan Infinity 100) and randomly captured.

The quantitative analysis of melanin was performed using ImageJ software version 1.53s (NIH). The black color was considered positive staining. This black color was extracted from the image, and the intensity of the image was quantified. Each group was compared to a control sample.

### 2.13. Statistical Analysis

The Kruskal–Wallis test was performed to compare the groups, followed by the Mann–Whitney U test for post hoc comparisons. The results were expressed as the mean ± standard deviation (SD). All statistical analyses were performed using SPSS version 26 (IBM, Armonk, NY, USA). Statistical significance is indicated in each figure legend.

## 3. Results

### 3.1. Characterization of EVE and PT Solutions

The TEM images of EVE solutions revealed round EVs with a bilayer membrane ranging between 30 nm and 150 nm in diameter (Figure S5A,B). The Western blot for plant EV markers (penetration 1; PEN1) [71] confirmed PEN1 expression in the EVE solution and also in the PT solution, albeit at a lower level in the latter (Figure S5C). Western blot also revealed the presence of NRF2 in the EVE solution and, at a lower level, in the PT solution (Figure S5D).

### 3.2. EVE Reduced the Expression of the Alpha-Melanocyte Stimulating Hormone (α-MSH) in Keratinocytes without Cytotoxicity

Cell viability assays showed no cytotoxic effects of PT at concentrations of up to 1000 μg/mL or EVE at concentrations of up to 3.0 × 10^10^ particles/mL (Figure S6). UV radiation increases the secretion of α-MSH from keratinocytes, which stimulates the cyclic adenosine monophosphate (cAMP)-PKA-cAMP response element-binding protein (CREB) axis and increases melanogenesis [72].

The expression of α-MSH in keratinocytes was increased by UV radiation and reduced by treatment with PT or EVE. The α-MSH expression was lowest in UV-radiated keratinocytes when PT was applied at concentrations of 50 μg/mL or 100 μg/mL or EVE was applied at concentrations of 1.5 × 10^8^ particles/mL or 3.0 × 10^8^ particles/mL, with no significant difference between these two doses for either substance (Figure S1B,C). 

We also evaluated which dosage of PT or EVE could decrease melanocyte proliferation most effectively. The melanocyte proliferation was lowest when PT was applied at concentrations of 50 μg/mL or 100 μg/mL or EVE was applied at concentrations of 1.5 × 10^8^ particles/mL or 3.0 × 10^8^ particles/mL, with no significant difference between these two doses for either substance (Figure S1E,F).

Therefore, 50 μg/mL of PT and 1.5 × 10^8^ particles/mL of the EVE solution were used as the optimal treatment concentration for further experiments.

### 3.3. PT and EVE Reduced Oxidative Stress in UV-Exposed Keratinocytes and Animal Skin

**Figure 1:**
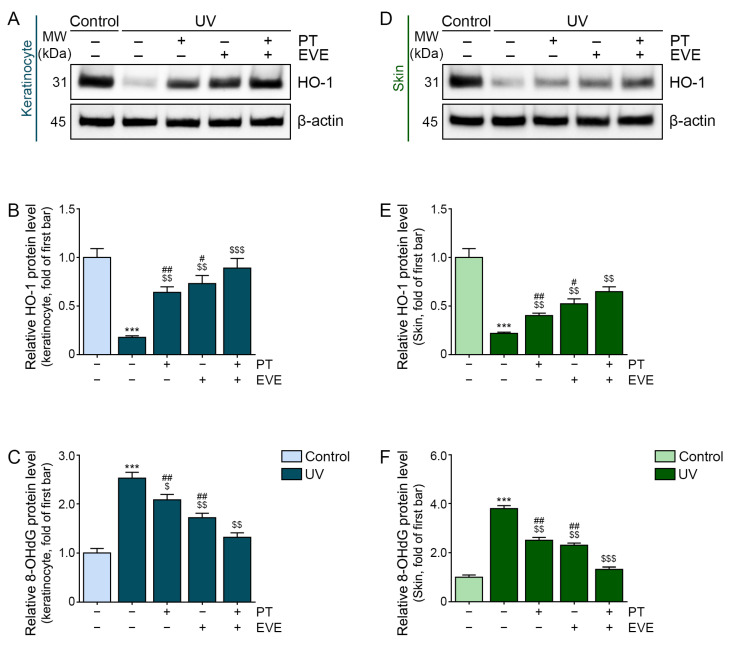
Regulation of oxidative stress by PT and EVE in UV-exposed keratinocytes and animal skin. (**A**,**B**) The protein expression of HO-1 in UV-exposed keratinocytes following PT and EVE treatment was measured by Western blot. (**C**) The protein expression of 8-OHdG in UV-exposed keratinocytes following PT and EVE treatment was measured by ELISA. (**D**,**E**) The protein expression of HO-1 in UV-exposed mouse skin following PT and EVE treatment was measured by Western blot. (**F**) The protein expression of 8-OHdG in UV-exposed mouse skin following PT and EVE treatment was measured by ELISA. Data are presented as the mean ± SD of three independent experiments. ***, *p* < 0.001, first bar vs. second bar; $, *p* < 0.05, $$, *p* < 0.01, and $$$, *p* < 0.001, second bar vs. third, fourth, fifth bar; #, *p* < 0.05 and ##, *p* < 0.01, fifth bar vs. third, fourth bar (Mann–Whitney U test). ELISA, enzyme-linked immunosorbent assay; EVE, extracellular vesicles from *E. cava*; HO-1, heme oxygenase-1; MW, molecular weight; PT, phlorotannin; SD, standard deviation; UV, ultraviolet; 8-OHdG, 8-hydroxy-2′-deoxyguanosine.

Keratinocytes were exposed to 30 s of UV radiation followed by treatment with PT, EVE, or PT and EVE (Figure S2). UV radiation reduced the expression of HO-1 in the keratinocytes, and this effect was reduced by both PT and EVE, with the PT and EVE co-treatment producing the greatest reduction in the HO-1 expression (Figure 1A,B). Oxidative stress was also evaluated on the basis of 8-hydroxy-2′-deoxyguanosine (8-OHdG) expression, which is frequently used as an oxidative stress marker [73]. UV radiation increased the 8-OHdG level in the keratinocytes, and this effect was reduced by both PT and EVE, with the PT and EVE co-treatment producing the strongest reduction in 8-OHdG levels (Figure 1C).

Melanin-possessing hairless mice were subjected to UV radiation and daily treatment with PT, EVE, or PT and EVE for 28 days (Figure S4). HO-1 expression in the skin of the mice was reduced by UV radiation, and this effect was reduced by both PT and EVE, with PT and EVE co-treatment producing the strongest increase in HO-1 levels (Figure 1D,E). Conversely, the 8-OHdG level in the skin of the mice was increased by UV radiation, and this effect was reduced by both PT and EVE, with PT and EVE co-treatment producing the greatest reduction in 8-OHdG levels (Figure 1F).

### 3.4. PT and EVE Reduced NLRP3 and IL-18 Levels in UV-Exposed Keratinocytes and Animal Skin

**Figure 2:**
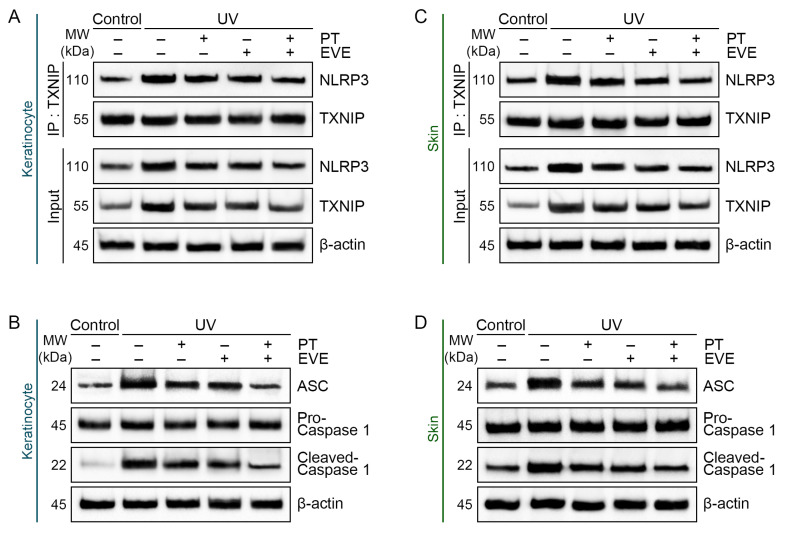

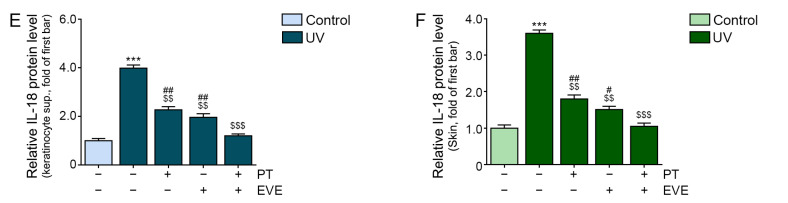
Regulation of NLRP3 inflammasomes and IL-18 by PT and EVE in UV-exposed keratinocytes and animal skin. (**A**) Co-immunoprecipitation and protein expression of NLRP3 and TXNIP in UV-exposed keratinocytes with or without PT and EVE treatments were measured by Western blot. (**B**) The protein expression of ASC, pro-caspase 1, and cleaved-caspase 1 in UV-exposed keratinocytes with or without PT and EVE treatments was measured by Western blot. (**C**) Co-immunoprecipitation and protein expression of NLRP3 and TXNIP in UV-exposed mouse skin with or without PT and EVE treatments were measured by Western blot. (**D**) The protein expression of ASC, pro-caspase 1, and cleaved-caspase 1 in UV-exposed mouse skin with or without PT and EVE treatments was measured by Western blot. (**E**) The protein expression of IL-18 in the UV-exposed supernatant of keratinocytes with or without PT and EVE treatments was measured by ELISA. (**F**) The protein expression of IL-18 in UV-exposed mouse skin with or without PT and EVE treatments was measured by ELISA. Data are presented as the mean ± SD of three independent experiments. ***, *p* < 0.001, first bar vs. second bar; $$, *p* < 0.01 and $$$, *p* < 0.001, second bar vs. third, fourth, fifth bar; #, *p* < 0.05 and ##, *p* < 0.01, fifth bar vs. third, fourth bar (Mann–Whitney U test). ASC, apoptosis-associated speck-like protein containing a caspase recruitment domain; ELISA, enzyme-linked immunosorbent assay; EVE, extracellular vesicles from *E. cava*; IL-18, interleukin-18; MW, molecular weight; NLRP3, nucleotide-binding oligomerization domain-like receptor family pyrin domain containing 3; PT, phlorotannin; SD, standard deviation; TXNIP, thioredoxin-interacting protein; UV, ultraviolet.

Co-IP assays showed that UV radiation increased binding between NLRP3 and TXNIP in keratinocytes, and this effect was reduced by PT and EVE, with PT and EVE co-treatment producing the strongest reduction (Figure 2A and Figure S7A). Western blot showed that UV radiation increased NLRP3 and TXNIP expression in UV-exposed keratinocytes, and this effect was reduced by PT and EVE, with PT and EVE co-treatment producing the strongest reduction (Figure 2A and Figure S7B,C). The expression of other NLRP3 inflammasome components (ASC and pro-caspase 1) and the activated form of caspase 1 (cleaved-caspase 1) were also evaluated in keratinocytes by Western blot. UV radiation increased the expression of ASC and the ratio of cleaved-caspase 1 to pro-caspase 1 (cleaved/pro-caspase 1), and these effects were reduced by PT and EVE, with PT and EVE co-treatment producing the strongest reductions (Figure 2B and Figure S7D,E). 

Similarly, UV radiation increased binding between NLRP3 and TXNIP in mouse skin, and this effect was reduced by PT and EVE, with PT and EVE co-treatment producing the strongest reduction (Figure 2C and Figure S8A). NLRP3 and TXNIP expression in mouse skin was increased by UV radiation, and these effects were reduced by PT and EVE, with PT and EVE co-treatment producing the strongest reduction (Figure 2C and Figure S8B,C). UV radiation also increased the expression of ASC and the ratio of cleaved/pro-caspase 1 in mouse skin, and these effects were reduced by PT and EVE, with PT and EVE co-treatment producing the strongest reductions (Figure 2D and Figure S8D,E).

ELISA was used to measure IL-18 secretion from keratinocytes and IL-18 levels in mouse skin. UV radiation increased IL-18 secretion by keratinocytes and IL-18 levels in mouse skin, and these effects were reduced by PT and EVE, with PT and EVE co-treatment producing the strongest reductions (Figure 2E,F).

### 3.5. PT and EVE Reduced Expression of NF-κB and MMPs in UV-Exposed Keratinocytes and Animal Skin

**Figure 3:**
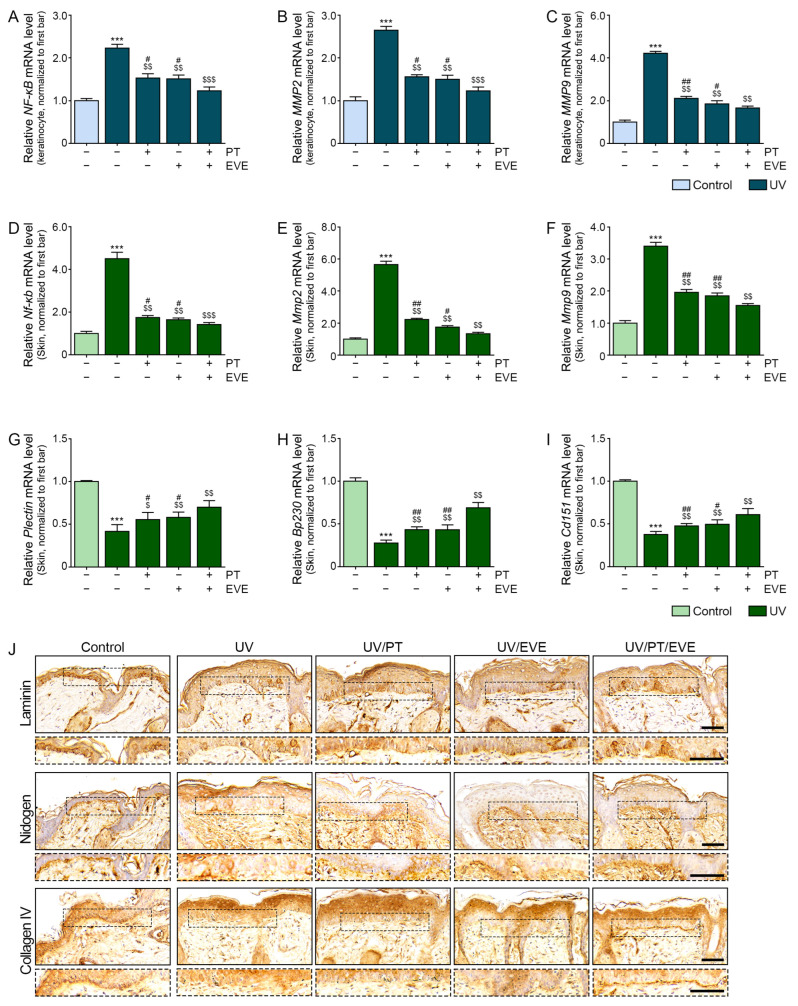
Regulation of NF-κB, MMPs, and BM components by PT and EVE in UV-exposed keratinocytes and animal skin. (**A**–**C**) The mRNA levels of NF-κB and MMP2/9 in UV-exposed keratinocytes with or without PT and EVE treatments were measured by qRT-PCR. (**D**–**F**) The mRNA levels of NF-κB and MMP2/9 in UV-exposed mouse skin with or without PT and EVE treatments were measured by qRT-PCR. (**G**–**I**) The mRNA levels of plectin, BP230, and CD151 in UV-exposed mouse skin with or without PT and EVE treatments were measured by qRT-PCR. (**J**) The protein expression of the BM components laminin, nidogen, and collagen type IV in UV-exposed mouse skin with or without PT and EVE treatments was measured by IHC. The dotted boxes are magnified image of the BM. Scale bar = 50 µm (black dashes). Data are presented as the mean ± SD of three independent experiments. ***, *p* < 0.001, first bar vs. second bar; $, *p* < 0.05, $$, *p* < 0.01 and $$$, *p* < 0.001, second bar vs. third, fourth, fifth bar; #, *p* < 0.05 and ##, *p* < 0.01, fifth bar vs. third, fourth bar (Mann–Whitney U test). BM, basement membrane; BP230, dystonin; EVE, extracellular vesicles from *E. cava*; IHC, immunohistochemistry; MMP, matrix metalloproteinase; NF-κB, nuclear factor kappa B; PT, phlorotannin; qRT-PCR, quantitative reverse-transcription polymerase chain reaction; SD, standard deviation; UV, ultraviolet.

UV radiation increased the mRNA expression of NF-κB and MMP2/9 in keratinocytes, and these effects were reduced by PT and EVE, with PT and EVE co-treatment producing the greatest reductions (Figure 3A–C). Similarly, UV radiation increased the mRNA expression of NF-κB and MMP2/9 in mouse skin, and these effects were reduced by PT and EVE, with PT and EVE co-treatment producing the greatest reductions (Figure 3D–F).

UV radiation reduced the mRNA expression of hemidesmosome components such as plectin, BP230, and CD151 in mouse skin, and these effects were reversed by PT and EVE, with PT and EVE co-treatment producing the strongest increases in expression (Figure 3G–I).

The expression of BM components (laminin, nidogen, and collagen type IV) was evaluated by immunohistochemistry staining in mouse skin. UV radiation reduced the expression of laminin, nidogen, and collagen type IV in mouse skin, and these effects were reversed by PT and EVE, with PT and EVE co-treatment producing the strongest increases in expression (Figure 3J and Figure S9).

### 3.6. PT and EVE Reduced Melanogenesis Pathway Signals in Melanocytes and UV-Exposed Animal Skin

Because PT and EVE were applied to the backs of mice using an MTS system, we assumed that keratinocytes of the epidermis would be directly exposed to PT or EVE and then secrete factors that cause melanocytes to reduce melanogenesis. We designed our in vitro model with UV-radiated keratinocytes and melanocytes accordingly. First, keratinocytes were exposed to UV radiation and treated with PT and/or EVE. Then, CM was collected from the keratinocyte cultures and used to treat melanocytes. We refer to CM from keratinocytes that were not irradiated and were subsequently treated with PBS as CM_PBS_. We refer to CM from irradiated keratinocytes that were subsequently treated with PBS as CM_UV_. We refer to CM from irradiated keratinocytes that were subsequently treated with PT, EVE, or PT and EVE as CM_PT_, CM_EVE_, and CM_PT/EVE_, respectively (Figure S3). 

**Figure 4:**
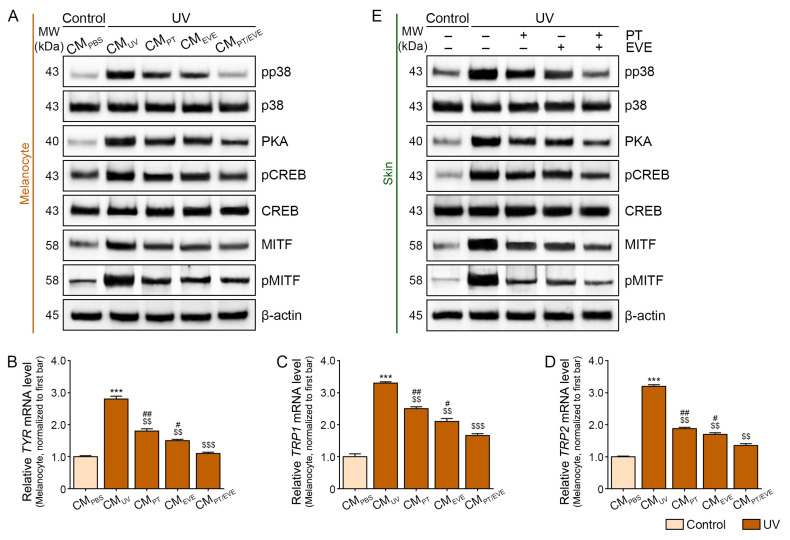

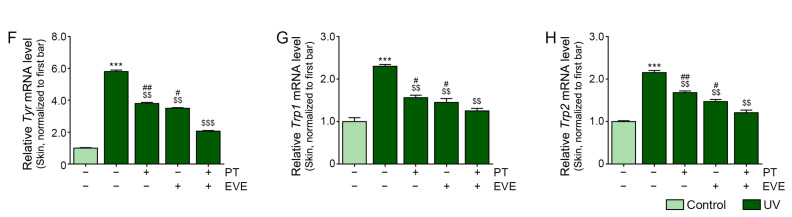
Regulation of melanogenesis pathway signals by PT and EVE in melanocytes and UV-exposed animal skin. (**A**) The protein expression of pp38, p38, PKA, pCREB, CREB, MITF, and pMITF in melanocytes treated with CM from UV-exposed keratinocytes with or without PT and EVE treatments was measured by Western blot. (**B**–**D**) The mRNA levels of TYR, TRP1, and TRP2 in melanocytes treated with CM from UV-exposed keratinocytes with or without PT and EVE treatments were measured by qRT-PCR. (**E**) The protein expression of pp38, p38, PKA, pCREB, CREB, MITF and pMITF in UV-exposed mouse skin with or without PT and EVE treatments was measured by Western blot. (**F**–**H**) The mRNA levels of TYR, TRP1, and TRP2 in melanocytes with or without PT and EVE treatments were measured by qRT-PCR. Data are presented as the mean ± SD of three independent experiments. ***, *p* < 0.001, first bar vs. second bar; $$, *p* < 0.01 and $$$, *p* < 0.001, second bar vs. third, fourth, fifth bar; #, *p* < 0.05 and ##, *p* < 0.01, fifth bar vs. third, fourth bar (Mann–Whitney U test). CM, conditioned media; CREB, cAMP response element-binding protein; EVE, extracellular vesicles from *E. cava*; MITF, microphthalmia-associated transcription factor; MW, molecular weight; p, phosphorated; PBS, phosphate-buffered saline; PKA, protein kinase A; pCREB, phosphorylated CREB; pMITF, phosphorylated MITF; PT, phlorotannin; pp38, phosphorylated p38; qRT-PCR, quantitative reverse–transcription polymerase chain reaction; SD, standard deviation; TRP, tyrosinase-related protein; TYR, tyrosinase; UV, ultraviolet.

After treating melanocytes with CM, we evaluated the ratio of phosphorated p38 to total p38 (pp38/p38), the expression of PKA, the ratio of phosphorated CREB to total CREB (pCREB/CREB), and the ratio of phosphorated MITF to total MITF (pMITF/MITF) by using Western blot. Each factor was increased in melanocytes treated with CM_UV_ compared to melanocytes treated with CM_PBS_, and these increases were reversed in melanocytes treated with CM_PT_, CM_EVE_, or CM_PT/EVE_, with CM_PT/EVE_ producing the most prominent reversals (Figure 4A and Figure S10A–D).

We also evaluated the mRNA expression of TYR, TRP1, and TRP2 in melanocytes treated with different CMs. The mRNA expression of all three proteins was higher in melanocytes treated with CM_UV_ compared to melanocytes treated with CM_PBS_, and these increases were reversed in melanocytes treated with CM_PT_, CM_EVE_, or CM_PT/EVE_, with the greatest reversals in melanocytes treated with CM_PT/EVE_ (Figure 4B–D).

Similarly, UV exposure increased pp38/p38, PKA expression, pCREB/CREB, and pMITF/MITF in mouse skin, and these effects were reduced by PT and EVE, with PT and EVE co-treatment producing the greatest reductions (Figure 4E and Figure S10E–H). UV radiation also increased the mRNA expression of TYR, TRP1, and TRP2 in mouse skin, and these effects were reduced by PT and EVE, with PT and EVE co-treatment producing the greatest reductions (Figure 4F–H).

### 3.7. PT and EVE Reduced Melanin Accumulation in UV-Exposed Animal Skin

**Figure 5:**
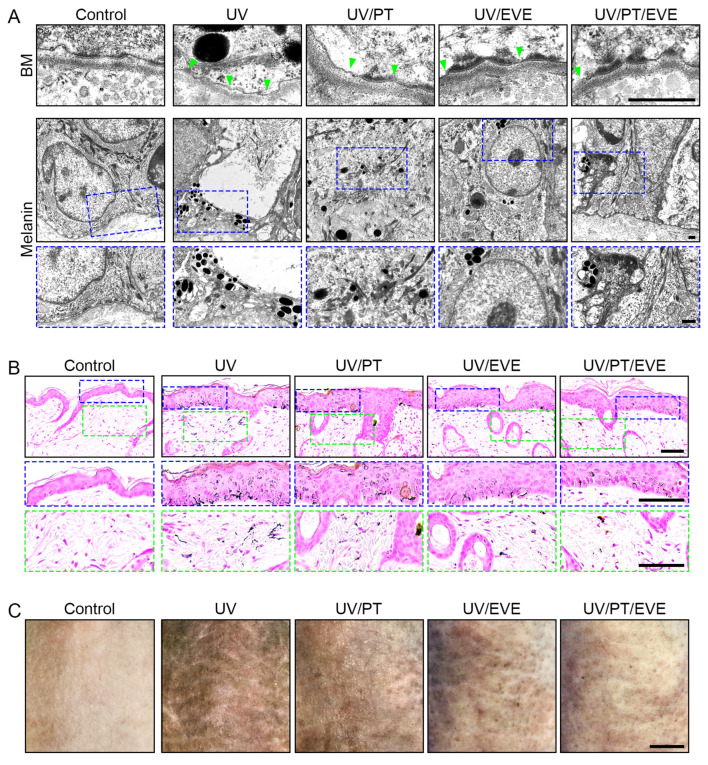
Regulation of melanin accumulation by PT and EVE treatments in UV-exposed animal skin. (**A**) The melanin and BM conditions were confirmed by TEM. The lamina densa (**upper**) and melanin content (**lower**) in TEM images were restored by PT and EVE treatments. The green mark represents lamina densa with disruptions or duplications (**upper**). The blue dotted boxes are magnified image of the lower TEM image. Scale bar = 500 nm (black dashes). (**B**) Melanin content was determined by Fontana Masson staining in the epidermis and dermis of mouse skin. The blue and green dotted boxes are magnified image of the fontana masson image. The blue dotted boxes are magnified image of epidermis, and the green boxes are magnified image of dermis. Scale bar = 100 µm (black dashes). (**C**) Skin lightness in UV-radiated mouse skin with or without PT and EVE treatments was measured. Scale bar = 500 µm (black dashes). BM, basement membrane; EVE, extracellular vesicles from *E. cava*; PT, phlorotannin; TEM, transmission electron microscopy; UV, ultraviolet.

UV radiation caused increased TYR activity in mouse skin, and PT and EVE reduced this effect, with PT and EVE co-treatments producing the greatest reduction (Figure S11A). The disruption of the lamina densa was evaluated by TEM. UV radiation increased the number of disrupted lesions in the lamina densa, and this effect was reduced by PT and EVE, with PT and EVE co-treatment producing the greatest reduction (Figure 5A upper and Figure S11B). UV radiation also increased the amount of melanosomes in TEM images of mouse skin, and this effect was reduced by PT and EVE, with PT and EVE co-treatment producing the greatest reduction (Figure 5A lower and Figure S11C). Melanin content in the mouse skin was evaluated by Fontana Masson staining separately in the epidermis and the dermis. UV radiation increased the melanin content in both the epidermis and the dermis, and these effects were reduced by PT and EVE, with PT and EVE co-treatment producing the greatest reductions (Figure 5B and Figure S11D,E). Skin lightness was evaluated with a CR-10 color reader. UV radiation increased skin lightness in the mouse skin, and this effect was reduced by PT and EVE, with PT and EVE co-treatment producing the greatest reduction (Figure 5C and Figure S11F).

## 4. Discussion

The administration of PT and/or EVE reduced UV-induced melanogenesis, oxidative stress, and levels of IL-18 and NLRP3 inflammasomes in keratinocytes and mouse skin. ROS increase NLRP3 inflammasome activation via increased binding between NLRP3 and TXNIP [23]. This, in turn, leads to increased IL-18 secretion, leading to NF-κB expression and the upregulation of melanogenesis pathway signals and MMPs that cause BM destruction [8,11,32,33,34,35,36]. Therefore, we hypothesized that reducing oxidative stress by treating keratinocytes and animal skin with EVE and/or PT would reduce UV-induced melanogenesis. 

PT isolated from *E. cava* has reported decreased ROS-induced tissue injuries via increasing NRF2 [74]. When cells are exposed to oxidative stress, NRF2 is translocated to the nucleus and binds to the antioxidant-responsive element (ARE), which increases various antioxidant enzymes such as HO-1, catalase (CAT), and superoxide dismutase (SOD) [75,76]. Melanogenesis is attenuated by increasing NRF2. Polysaccharide, isolated from *Cistanche deserticola*, is reported to decrease melanogenesis by increasing the NRF2/HO-1 pathway [77]. Since PT is known to increase the NRF2/HO-1 pathway, which can decrease melanogenesis, we thought that PT or EVE could decrease melanogenesis via the NRF2 pathway.

We first determined whether our PT and EVE solutions contained NRF2, which has powerful antioxidant effects [27]. Western blots showed that NRF2 was present in both solutions, and the NRF2 concentration was higher in the EVE solution than in the PT solution. In previous studies, EVs derived from adipose-derived stem cells contained NRF2 [78], and various pEVs derived from carrot or lemon increased the expression of NRF2 and HO-1 [79,80]. 

Since EVE contains NRF2 and PT is well known to increase the NRF2/HO-1 pathway, we assumed that the co-treatment of PT and EVE could have a synergistic effect on attenuating melanogenesis via the upregulation of HO-1. The α-MSH expression was most prominently decreased when PT or EVE were treated at concentrations of 50 μg/mL or 1.5 × 10^8^ particles/mL, respectively. Melanocyte proliferation also prominently decreased at concentrations of 50 μg/mL or 1.5 × 10^8^ particles/mL, respectively. Thus, we evaluated the co-treatment effect at these concentrations. EVE expressed more NRF2 than PT solutions; thus, the anti-pigmentation effect could differ by the dosage ratio between PT and EVE. The dosage ratio which could maximize the anti-pigmentation effect should be evaluated in future studies.

To determine if PT or EVE could reduce oxidative stress, we performed Western blot and ELISA to measure the levels of HO-1 and 8-OHdG, respectively, in UV-irradiated keratinocytes and mouse skin. The levels of both proteins were reduced by PT and EVE after UV irradiation, suggesting that PT and EVE reduced UV-induced oxidative stress.

Next, we evaluated whether reduced oxidative stress affected NLRP3 inflammasome activation. UV-exposed keratinocytes showed increased binding between NLRP3 and TXNIP, which was accompanied by the increased expression of the NLRP3 inflammasome components NLRP3, ASC, and pro-caspase-1. PT and EVE reduced these effects. Therefore, we assumed that NRF2 might be one of the biomolecules that reduce melanogenesis. Melanogenesis was most effectively reduced when PT and EV were simultaneously applied in both the in vitro study and the animal study. Thus, co-treatment with PT and EVE was the most effective way to reduce melanogenesis.

Our results are in line with those of previous studies showing that a reduction in ROS levels suppressed TXNIP-induced NLRP3 inflammasome activation. The activation of ROS/TXNIP/NLRP3-related pathways was reported in various skin diseases and in skin injury in trichloroethylene-sensitized mice [81]. TXNIP was also reported to activate NLRP3 inflammasomes in inflammation-related diseases, such as osteoarthritis [82]. Ceramide-induced oxidative stress increased TXNIP/NLRP3 pathway activation and IL-18 expression in endothelial cells; however, the secretion of IL-18 was reduced by treatment with the antioxidant N-acetyl-L-cysteine (NAC) [83]. Treatment with the TXNIP inhibitor verapamil or siRNA-targeting TXNIP reduced NLRP3 inflammasome activation [84]. In one study in which UV exposure led to increased ROS levels and NLRP3 inflammasome activation in keratinocytes, NLRP3 inflammasome activation was reduced by polyphenols such as rosmarinic acid and fucoxanthin that activated NRF2 and HO-1 [85]. 

IL-18 secretion is increased by NLRP3 inflammasome activation [14]. In co-cultures of keratinocytes and melanocytes, UV radiation increased IL-18 secretion in keratinocytes and melanogenesis in melanocytes, and the treatment of melanocytes (B16F10 and NHEM) with IL-18 led to dose-dependent increases in TYR activity [11]. IL-18 also increases MITF, TYRP1, and TYRP2 expression via the upregulation of NF-κB in melanocytes [8]. In line with previous studies, our experiments showed that IL-18 secretion by keratinocytes was increased by UV radiation and reduced by the administration of PT and EVE.

We treated melanocytes with CM from keratinocytes to determine if increased IL-18 secretion by keratinocytes affects melanogenesis. CM from UV-exposed keratinocytes increased melanogenesis by inducing the p38, PKA, CREB, MITF, TYR, TRP1, and TRP2 pathways in melanocytes. However, these effects were reduced when the UV-exposed keratinocytes were treated with PT and/or EVE. These results suggest that PT and EVE can suppress IL-18 secretion from keratinocytes and, thus, reduce the expression of melanogenesis-related signal pathways in melanocytes. Similar to the in vitro results, UV exposure reduced HO-1 levels and increased 8-OHdG levels, NLRP3 inflammasome activation, TXNIP expression, and IL-18 expression in mouse skin. Treatment with PT and/or EVE reversed these effects and also reduced p38, PKA, CREB, MITF, TYR, TRP1, and TRP2 expression. 

UV radiation induces NF-κB activation, which eventually activates MMPs [86,87]. *E. cava* extract was previously shown to reduce the effect of NLRP3/NF-κB pathway activation and inflammation in colitis [68]. In another study, dioxinodehydroeckol from *E. cava* inhibited the proliferation of MCF-7 breast cancer cells by modulating the NF-κB signaling pathway [88]. Another PT, 6,6′-bieckol, inhibited NF-κB and MMP2/9 expression, which eventually reduced cancer cell migration [89]. We found that PT and EVE reduced NF-κB and MMP2/9 expression in UV-exposed animal skin, and these reductions were accompanied by increased levels of the BM components laminin, nidogen, and collagen type IV.

Hemidesmosomes consist of BP230, CD151, plectin, integrin α6β4, and collagen XVII (also called BP180) and have a role in increasing the attachment between keratinocytes and BM [90,91]. MMPs also destroy hemidesmosome proteins [92]. The expressions of plectin, BP230, and CD151 were increased by UV radiation and decreased by PT or EVE. BM is a sheet-like structure which mainly consists of laminin, nidogen, and collagen type IV [93,94].

In a previous study, the BM of UV-exposed skin showed disruption at the dermal–epidermal junction and reduplicated lamina densa [95,96]. Conversely, the UV-induced destruction of BM was repaired by the inhibition of MMP or heparanase [96,97,98,99]. Moreover, BM regeneration was promoted by increasing collagen IV and laminin 332 expression [100,101]. BM damage has been found in 95.8% of hyperpigmented areas such as melasmas [102]. Because BM disruption allows the migration of melanocytes, UV-induced BM disruption is related to the induction of dermal pigmentation [43]. Pendulous melanocytes, which have migrated into the dermis, are frequently observed in hyperpigmented lesions such as melasmas, café-au-lait spots, and senile lentigos [103,104,105,106]. In our study, UV exposure caused the disruption of the lamina densa, and this disruption was reduced by treatment with PT and/or EVE. Moreover, the epidermal and dermal melanin content in UV-exposed skin was reduced when the skin was treated with PT and/or EVE. 

Dermal pigmentation is more resistant than epidermal pigmentation to local treatments such as creams. Melasma is especially hard to treat, and outcomes are often unsatisfactory because of frequent relapses and complications [107]. For example, treatment with 4% hydroquinone results in the improvement of hyperpigmentation in 77% of patients [108]; however, 25% of patients experience dose-dependent and duration-dependent irritation [109,110]. Moreover, prolonged treatment, or treatment with higher concentrations of hydroquinone, can cause irritation and allergic dermatitis, hypopigmentation, erythema, exogenous ochronosis, and burning sensations [108,109,110,111]. Because PT and EVE reduce dermal melanogenesis as well as epidermal melanogenesis, these substances might be effective treatments for dermal melanogenesis. Since we did not compare the anti-melanogenesis effect of PT and EVE with hydroquinone in this study, we cannot definitively suggest whether EVE or PT is more effective than hydroquinone. The superiority of EVE or PT should be evaluated in future studies. We evaluated the effect of EVE or PT on melanogenesis for 28 days. Thus, the long-term effects of EVE or PT should be evaluated in humans as a future study.

EVs are absorbable through the skin, but the absorption is less than 1% when the EVs are topically applied because absorption is hindered by the stratum granulosum [110]. Therefore, to increase the absorption of EVE, we used an MTS system, which is known to efficiently deliver target substances into dermal tissue through the stratum corneum barrier [112].

## 5. Conclusions

EVE contains NRF2 and effectively reduces oxidative stress and TXNIP/NRLP3 inflammasome activation when it is applied to UV-exposed keratinocytes and animal skin. This is accompanied by reduced IL-18 secretion and the reduced expression of melanogenesis signaling pathways. In UV-exposed animal skin, EVE also caused reductions in MMP2/9 levels and increases in collagen IV, nidogen, and laminin levels, which were accompanied by reductions in both epidermal and dermal melanin accumulation.

## Data Availability

All data are contained within the article and Supplementary Materials.

## Supplementary Materials

The following supporting information can be downloaded at: https://www.mdpi.com/article/10.3390/antiox13040408/s1, Figure S1: Optimization of PT and EVE concentrations in vitro; Figure S2: Schematic of keratinocytes with UV-induced pigmentation for the evaluation of PT and EVE; Figure S3: Schematic of melanocytes affected by keratinocytes with UV-induced pigmentation for the evaluation of PT and EVE; Figure S4: Schematic of mice with UV-induced pigmentation for the evaluation of PT and EVE; Figure S5: TEM image of EVE and confirmation of surface marker and NRF2 expression in EVE and PT; Figure S6: In vitro cytotoxic effects of PT and EVE treatments; Figure S7: Regulation of NLRP3 inflammasome by PT and EVE treatments in UV-exposed keratinocytes; Figure S8: Regulation of NLRP3 inflammasome by PT and EVE treatments in UV-exposed animal skin; Figure S9: Regulation of BM components by PT and EVE in UV-exposed animal skin; Figure S10: Regulation of melanogenesis pathway signals by PT and EVE in melanocytes and UV-exposed animal skin; Figure S11: Regulation of melanin accumulation by PT and EVE treatments in UV-exposed animal skin; Table S1: Concentration analysis of EVE used in this study; Table S2: List of primers for quantitative reverse–transcription polymerase chain reaction.; Table S3: List of antibodies for Western blot (WB), ELISA, and IHC.
