# Supplementary material for: Co-Treatment with Phlorotannin and Extracellular Vesicles from Ecklonia cava Inhibits UV-Induced Melanogenesis
Source: Antioxidants (Basel). 2024 Mar 28;13(4):408. doi: 10.3390/antiox13040408 (PMC11047619; doi:10.3390/antiox13040408)
Supplement: Supplementary file 1 [file antioxidants-13-00408-s001.zip › antioxidants-2913923-supplementary.pdf]

antioxidants

Supplementary Materials

Article

Co-Treatment with Phlorotannin and Extracellular Vesicles from *Ecklonia cava* Inhibits UV-Induced Melanogenesis

Kyung-A Byun [1,2,3,†], Youngjin Park [4,†], Seyeon Oh [3], Sosorburam Batsukh [1,3], Kuk Hui Son [5,*] and Kyunghee Byun [1,3,6,*]

[1] Department of Anatomy & Cell Biology, College of Medicine, Gachon University, Incheon 21936, Republic of Korea
[2] LIBON Inc., Incheon 22006, Republic of Korea
[3] Functional Cellular Networks Laboratory, Lee Gil Ya Cancer and Diabetes Institute, Gachon University, Incheon 21999, Republic of Korea
[4] OBLIV CLINIC, Incheon 21998, Republic of Korea
[5] Department of Thoracic and Cardiovascular Surgery, Gachon University Gil Medical Center, Gachon University, Incheon 21565, Republic of Korea
[6] Department of Health Sciences and Technology, Gachon Advanced Institute for Health & Sciences and Technology (GAIHST), Gachon University, Incheon 21999, Republic of Korea
[*] Correspondence: dr632@gachon.ac.kr (K.H.S.); khbyun1@gachon.ac.kr (K.B.); Tel.: +82-32-460-3666 (K.H.S.); +82-32-899-6511 (K.B.)
[†] These authors contributed equally to this work.

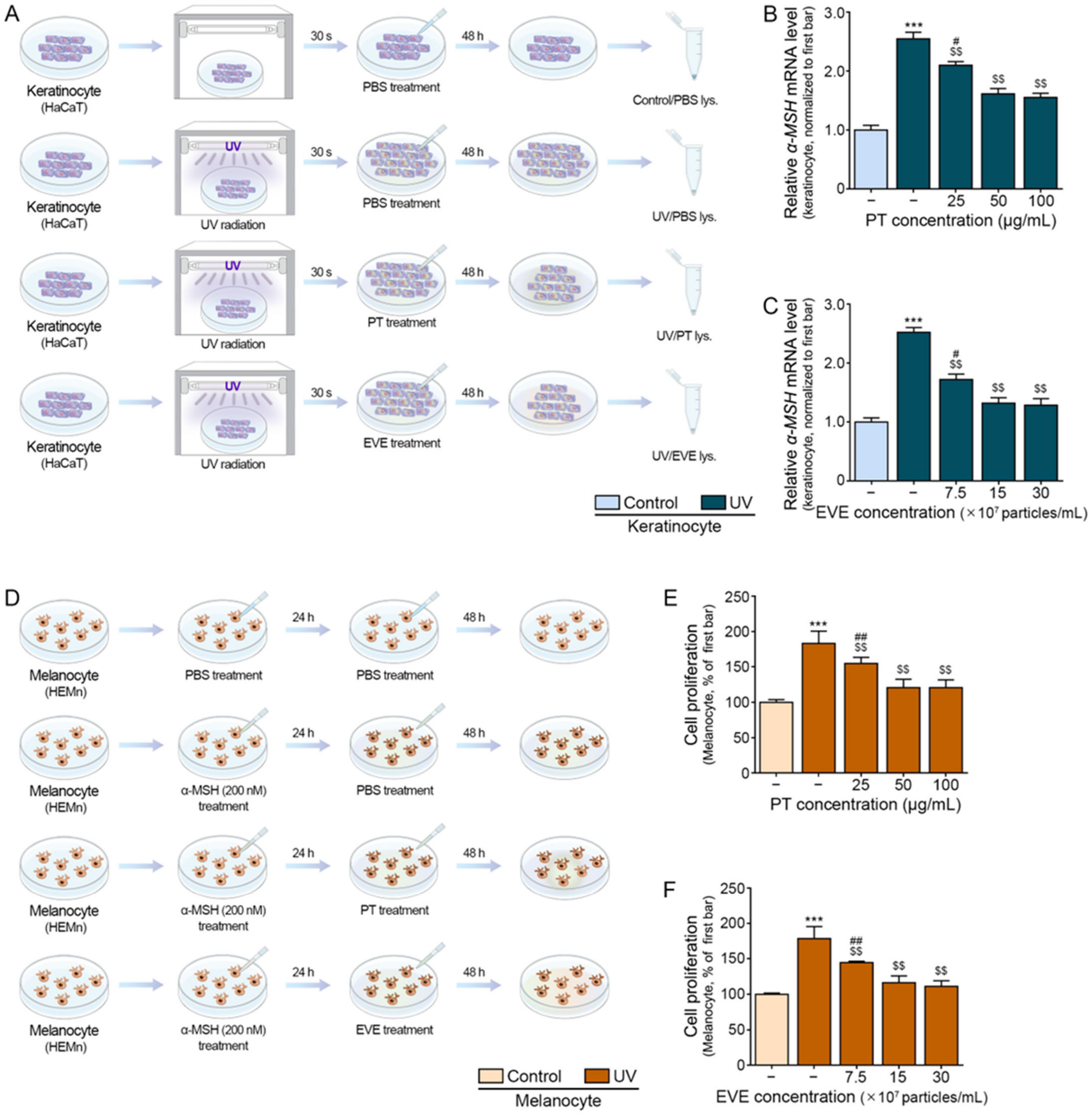

Figure S1. Optimization of PT and EVE concentrations *in vitro*. (**A**) Schematic diagram of the treatment of UV-exposed keratinocytes with PT or EVE. (**B**,**C**) The mRNA levels of α-MSH in UV-exposed keratinocytes following PT (**B**) or EVE (**C**) treatment were measured by qRT-PCR. (**D**) Schematic diagram of the treatment of melanocyte with PT or EVE. (**E**,**F**) Cell proliferation was assessed in melanocytes following PT (**E**) or EVE (**F**) treatment by CCK-8 assay. Data are presented as the mean ± SD of three independent experiments. ***, $p < 0.001$, first bar vs. second bar; $$, $p < 0.01$, second bar vs. third, fourth, fifth bar; #, $p < 0.05$ and ##, $p < 0.01$, fifth bar vs. third, fourth bar (Mann–Whitney U test). α-MSH, α-melanocyte-stimulating hormone; EVE, extracellular vesicles from *E. cava.*; PBS, phosphate-buffered saline; PT, phlorotannin; qRT-PCR, quantitative reverse–transcription polymerase chain reaction; SD, standard deviation; UV, ultraviolet.

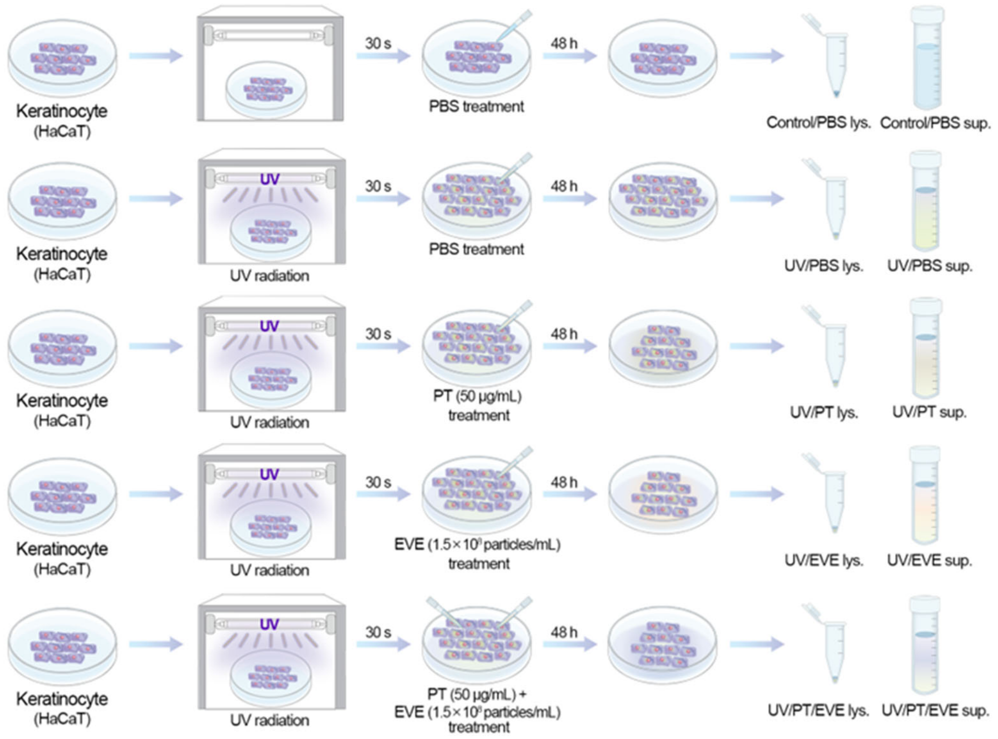

Figure S2. Schematic of keratinocytes with UV-induced pigmentation for the evaluation of PT and EVE. EVE, extracellular vesicles from *E. cava*; lys, lysate; PBS, phosphate-buffered saline; PT, phlorotannin; sup, supernatant; UV, ultraviolet.

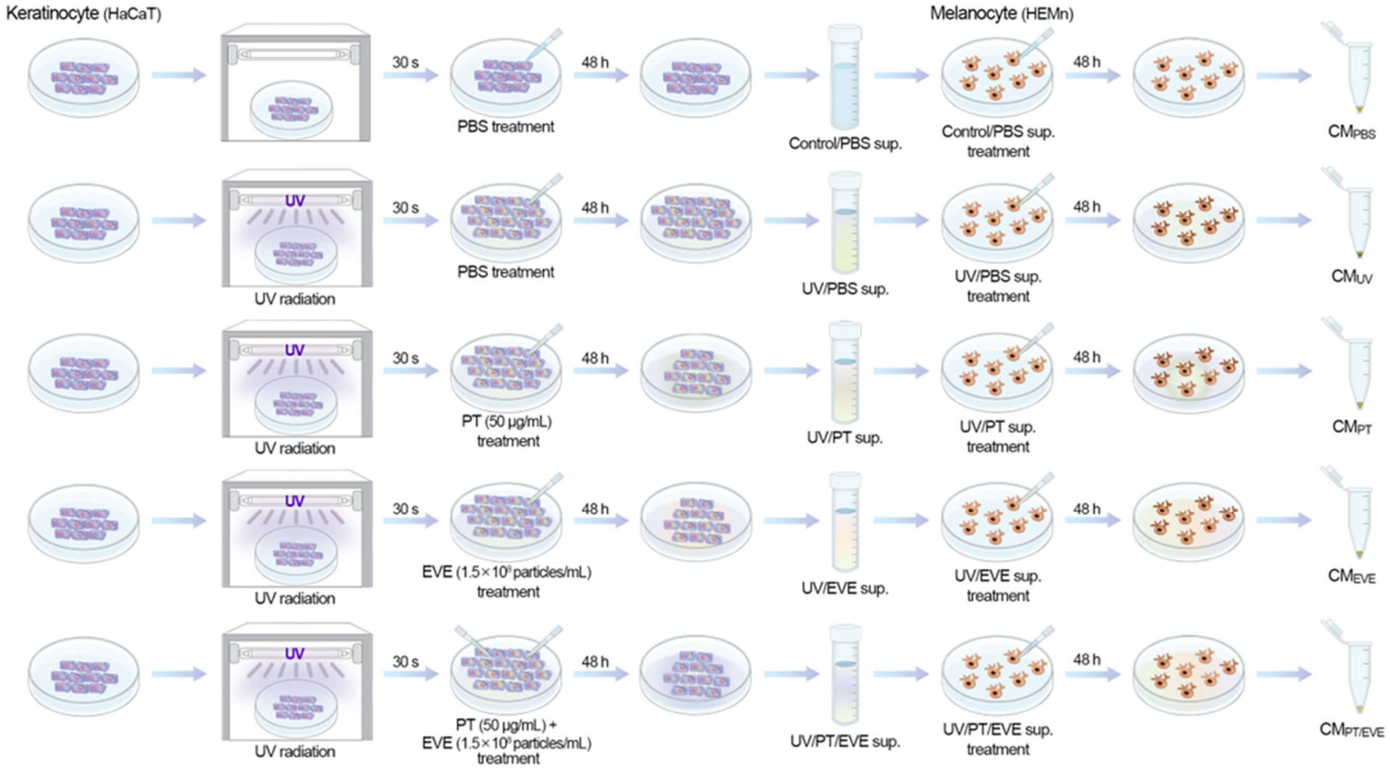

Figure S3. Schematic of melanocytes affected by keratinocytes with UV-induced pigmentation for the evaluation of PT and EVE. CM, conditioned media from keratinocyte; EVE, extracellular vesicles from *E. cava*; PBS, phosphate-buffered saline; PT, phlorotannin; sup, supernatant; UV, ultraviolet.

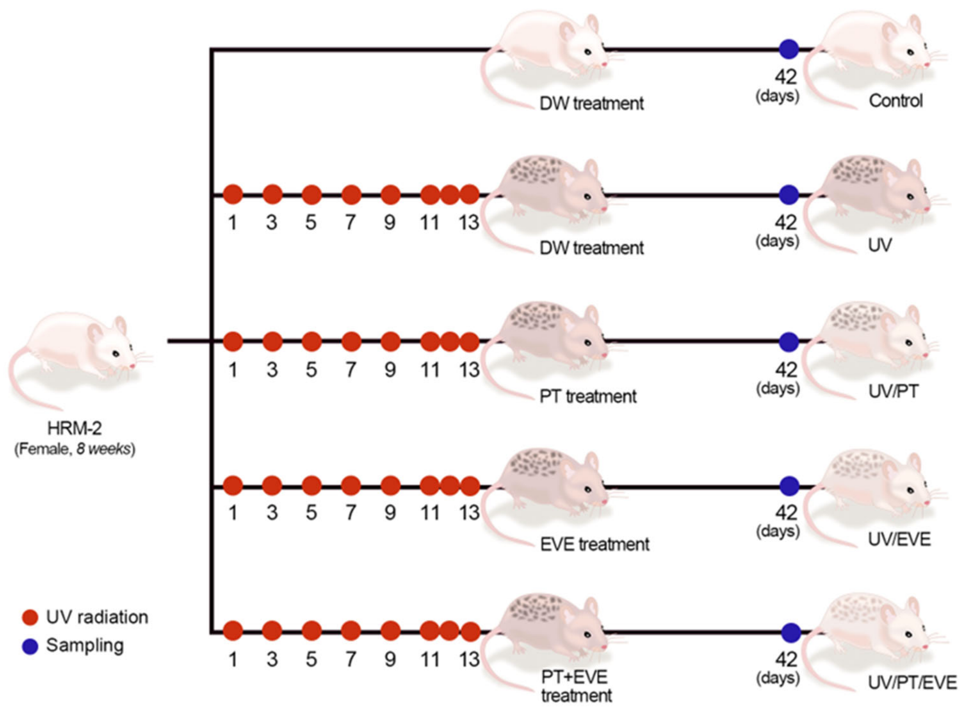

Figure S4. Schematic of mice with UV-induced pigmentation for the evaluation of PT and EVE. On 2 weeks after UV radiation starting, PBS, PT (concentration of 1 mg/mL), EVE (concentration of 3.0×10^{10} particles/mL), or mixture of PT and EVE (each of concentration of 1 mg/mL and 3.0×10^{10} particles/mL) was administered into animal skin with micro needling system. Total injection volume of all solution was 200 µL. After 28 days of injection, animal skin was harvested. DW, distilled water; EVE, extracellular vesicles from *E. cava*; PT, phlorotannin; UV, ultraviolet.

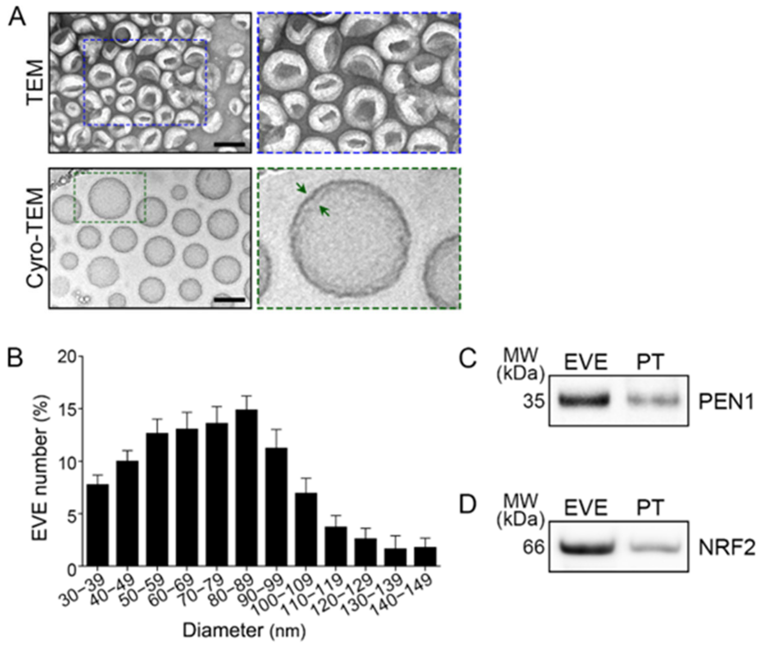

Figure S5. (A) TEM image of EVE and confirmation of surface marker and NRF2 expression in EVE and PT. Blue dotted lines indicate a magnified image of negative staining. Green dotted lines represent a magnified image of cryo-TEM, and the green arrows show a lipid bilayer membrane of a vesicle. Scale bar= 100 nm. **(B)** Quantitative assessment of EVE diameter based on TEM images. **(C,D)** Western blot analysis of plant EV markers PEN1 (**C**) and NRF2 (**D**) in PT and EVE. EVE, extracellular vesicles from *E. cava*; MW, molecular weight; NRF2, nuclear factor erythroid-2-related factor 2; PEN1, penetration 1; PT, phlorotannin; TEM, transmission electron microscopy.

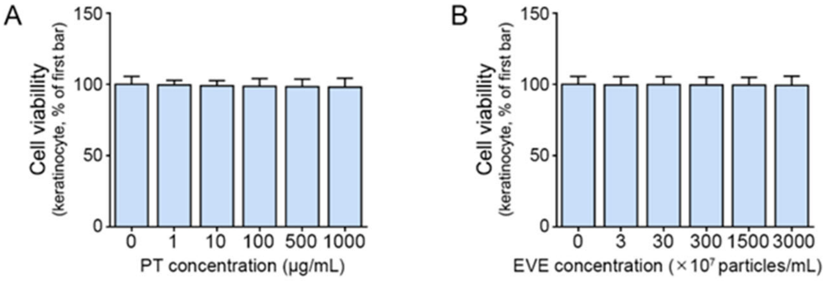

Figure S6. *In vitro* cytotoxic effects of PT and EVE treatments. (**A**,**B**) The cell viability of keratinocytes following PT (**A**) or EVE (**B**) treatment was measured. Data are presented as the mean ± SD of three independent experiments. EVE, extracellular vesicles from *E. cava*; PT, phlorotannin; SD, standard deviation.

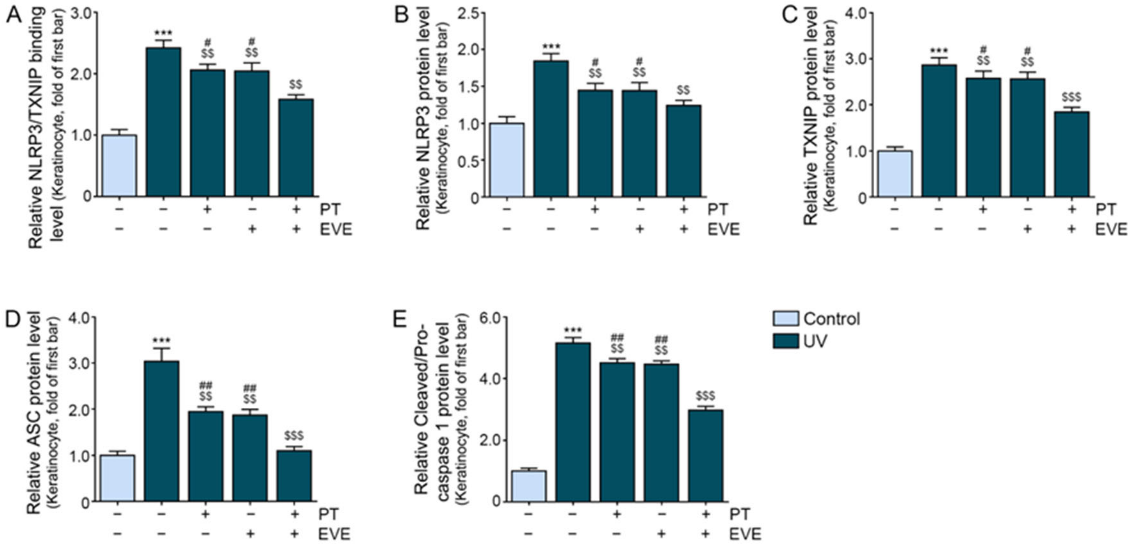

Figure S7. Regulation of NLRP3 inflammasome by PT and EVE treatments in UV-exposed keratinocytes. (**A**–**C**) Quantitative assessment of Western blot data presented in Figure 2A. (**D**,**E**) Quantitative assessment of Western blot data presented in Figure 2B. Data are presented as the mean ± SD of three independent experiments. ***, $p < 0.001$, first bar vs. second bar; $$, $p < 0.01$ and $$$, $p < 0.001$, second bar vs. third, fourth, fifth bar; #, $p < 0.05$ and ##, $p < 0.01$, fifth bar vs. third, fourth bar (Mann–Whitney U test). ASC, apoptosis-associated speck-like protein containing a caspase recruitment domain; EVE, extracellular vesicles from *E. cava*; NLRP3, nucleotide-binding oligomerization domain-like receptor family pyrin domain containing 3; PT, phlorotannin; SD, standard deviation; TXNIP, Thioredoxin-interacting protein; UV, ultraviolet.

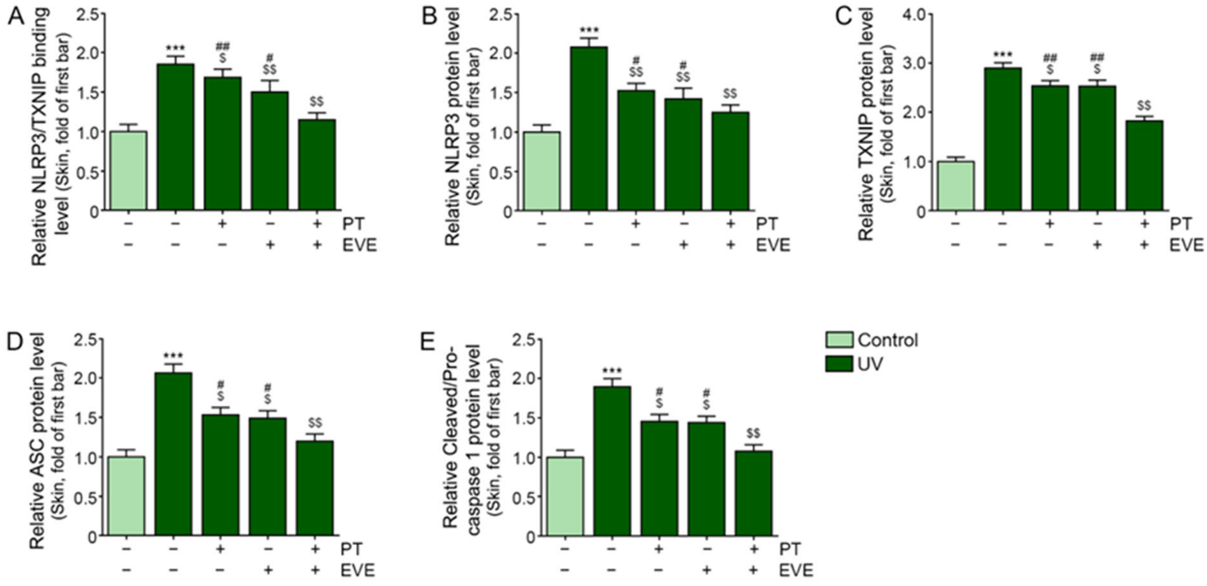

Figure S8. Regulation of NLRP3 inflammasome by PT and EVE treatments in UV-exposed animal skin. (**A–C**) Quantitative assessment of Western blot data presented in Figure 2C. (**D,E**) Quantitative assessment of Western blot data presented in Figure 2D. Data are presented as the mean ± SD of three independent experiments. ***, $p < 0.001$, first bar vs. second bar; $, $p < 0.05$ and $$, $p < 0.01$, second bar vs. third, fourth, fifth bar; #, $p < 0.05$ and ##, $p < 0.01$, fifth bar vs. third, fourth bar (Mann–Whitney U test). ASC, apoptosis-associated speck-like protein containing a caspase recruitment domain; EVE, extracellular vesicles from *E. cava*; NLRP3, nucleotide-binding oligomerization domain-like receptor family pyrin domain containing 3; PT, phlorotannin; SD, standard deviation; TXNIP, Thioredoxin-interacting protein; UV, ultraviolet.

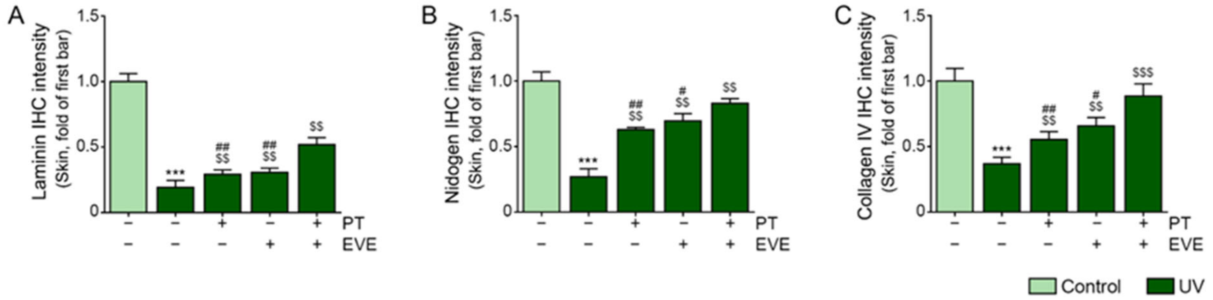

Figure S9. Regulation of BM components by PT and EVE in UV-exposed animal skin. (**A–C**) Quantitative assessment of IHC presented in Figure 3J. Data are presented as the mean ± SD of three independent experiments. ***, $p < 0.001$, first bar vs. second bar; $$, $p < 0.01$ and $$$, $p <$ 0.001, second bar vs. third, fourth, fifth bar; #, $p < 0.05$ and ##, $p < 0.01$, fifth bar vs. third, fourth bar (Mann–Whitney U test). BM, basement membrane; EVE, extracellular vesicles from *E. cava*; IHC, immunohistochemistry; PT, phlorotannin; SD, standard deviation; UV, ultraviolet.

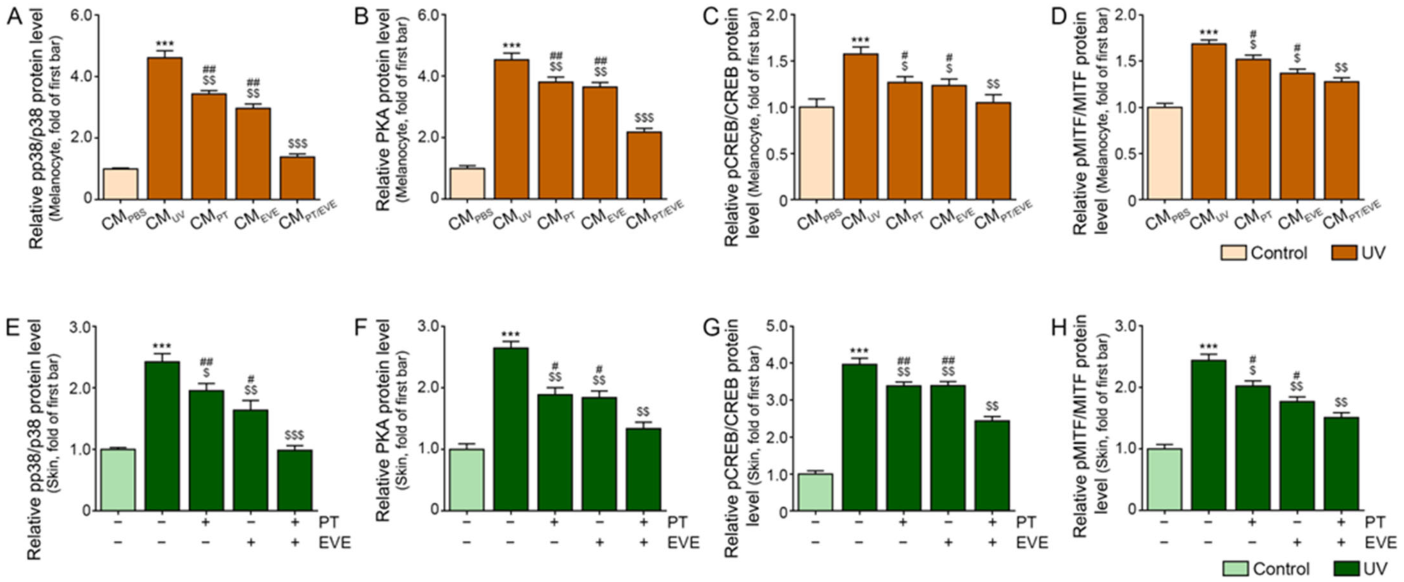

Figure S10. Regulation of melanogenesis pathway signals by PT and EVE in melanocytes and UV-exposed animal skin. (**A–H**) Quantitative assessment of Western blot data presented in Figure 4A (**A–D**) and Figure 4E (**E–H**). Data are presented as the mean ± SD of three independent experiments. ***, $p < 0.001$, first bar vs. second bar; $, $p < 0.05$, $$, $p < 0.01$, and $$$, $p < 0.001$, second bar vs. third, fourth, fifth bar; #, $p < 0.05$ and ##, $p < 0.01$, fifth bar vs. third, fourth bar (Mann–Whitney U test). CM, conditioned media; CREB, cAMP response element binding protein; EVE, extracellular vesicles from *E. cava*; MITF, microphthalmia-associated transcription factor; p, phosphorated; PBS, phosphate-buffered saline; PKA, protein kinase A; pCREB, phosphorylated CREB; pMITF, phosphorylated MITF; PT, phlorotannin; pp38, phosphorylated p38; SD, standard deviation; UV, ultraviolet.

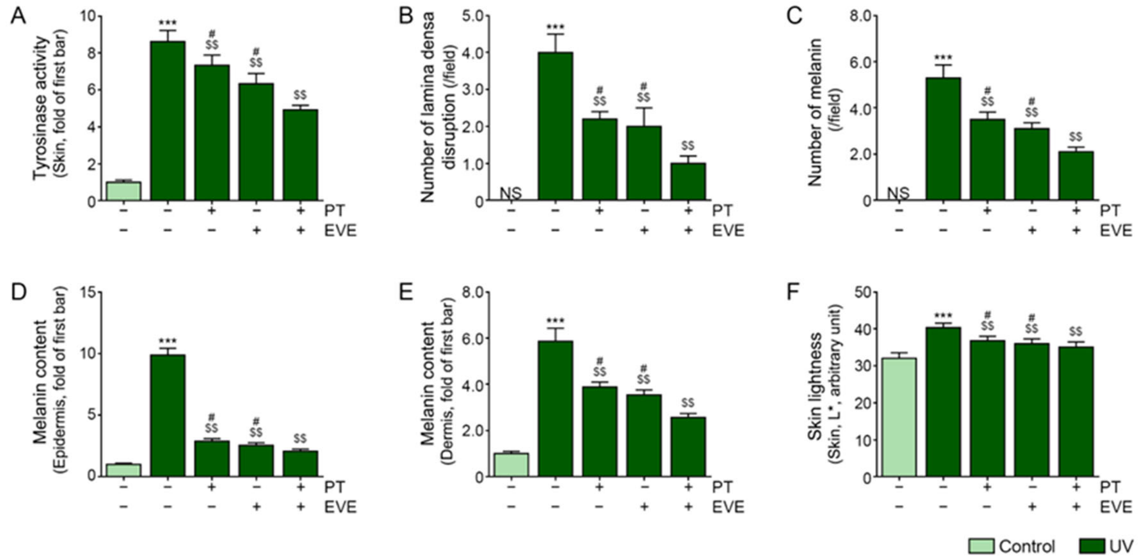

Figure S11. Regulation of melanin accumulation by PT and EVE treatments in UV-exposed animal skin. (**A**) The tyrosinase activity in UV-exposed mouse skin following PT and EVE treatment was measured. (**B,C**) Quantitative assessment of TEM images presented in Figure 5A. (**D,E**) Quantitative assessment of Fontana Masson staining in the epidermis and dermis of mouse skin presented in Figure 5B. (**F**) Quantitative assessment of skin lightness measured using a CR-10 color reader presented in Figure 5C. Data are presented as the mean ± SD of three independent experiments. ***, $p < 0.001$, first bar vs. second bar; $$, $p < 0.01$, second bar vs. third, fourth, fifth bar; #, $p < 0.05$, fifth bar vs. third, fourth bar (Mann–Whitney U test). EVE, extracellular vesicles from *E. cava*; NS, non-signals; PT, phlorotannin; SD, standard deviation; TEM, transmission electron microscopy; UV, ultraviolet.

Table S1. Concentration analysis of EVE used in this study.

Initial *E. cava* material		12 g
NTA	Diluent	2000
	dilution concentration	$1.82 \pm 0.15 \times 10^8$ particles/mL
	Total concentration	$3.64 \pm 0.29 \times 10^{11}$ particles/mL
	Concentration per 1 g of *E. cava*	$3.03 \pm 0.24 \times 10^{10}$ particles/mL
BCA	Concentration	11.8 mg/mL
	Concentration per 1 g of *E. cava*	0.98 mg/mL

BCA, bicinchoninic acid assay; EVE, extracellular vesicles from *E. cava*; NTA; nanoparticle-tracking analysis.

Table S2. List of primers for quantitative reverse–transcription polymerase chain reaction.

Accession Number	Gene (Organism)	Primer Sequences	
NM_001101.5	*ACTB (human)*	Forward	5'-CTC GCC TTT GCC GAT CC-3′
		Reverse	5'-TCT CCA TGT CGT CCC AGT TG-3′
NM_003998.4	*NF-κB (human)*	Forward	5'-ACC TAG CTG CCA AAG AAG GAC-3′
		Reverse	5'-TGG GGT GGT CAA GAA GTA GTG-3′
NM_004530.6	*MMP2 (human)*	Forward	5'-CAC TTT CCT GGG CAA CAA ATA-3′
		Reverse	5'-CTT GCG GTC ATC ATC GTA GTT-3′
NM_004994.3	*MMP9 (human)*	Forward	5'-AAA GCC TAT TTC TGC CAG GAC-3′
		Reverse	5'-TCA TAG GTC ACG TAG CCC ACT-3′
NM_000372.5	*TYR (human)*	Forward	5'-TGT TTT GTA CTG CCT GCT GTG-3′
		Reverse	5'-AGC ATT CCT TCT CCA TCA GGT-3′
NM_000550.3	*TRP1 (human)*	Forward	5'-GTG CCA CTG TTG AGG CTT TG-3′
		Reverse	5'-ATG GGG ATA CTG AGG GCT GT-3′
NM_001922.5	*TRP2 (human)*	Forward	5'-AAA GCC TGA CTT AAC GGG GG-3′
		Reverse	5'-GGA TTT TGC AGC CCA AGC AA-3′
NM_000939.4	*α-MSH (human)*	Forward	5'-GTT TCA TGA CCT CCG AGA AGA G-3′
		Reverse	5'-CTT GTA GGC GTT CTT GAT GAT G-3′
NM_007393.5	*Actb (mouse)*	Forward	5'-CCG TAA AGA CCT CTA TGC CAA C-3′
		Reverse	5'-GCA GTA ATC TCC TTC TGC ATC C-3′
NM_008689.3	*Nf-κb (mouse)*	Forward	5'-CAG TGG TGT GGA GAC ATC CTT-3′
		Reverse	5'-TCA GAG ATA GCA GTG GGC TGT-3′
NM_008610.3	*Mmp2 (mouse)*	Forward	5'-GAT GAT GAC CGG AAG TGG GG-3′
		Reverse	5'-ATG AAG ATG ATA GGG CCC GTG-3′
NM_013599.5	*Mmp9 (mouse)*	Forward	5'-GGG TCT AGG CCC AGA GGT AA-3′
		Reverse	5'- TAA CGC CCA GTA GAG AGC CT-3′
NM_011661.6	*Tyr (mouse)*	Forward	5'-CAG CAT CCT TCT TCT CCT CCT-3′
		Reverse	5'-GTT CCA TCG CAT AAA ACC TGA-3′
NM_031202.3	*Trp1 (mouse)*	Forward	5'-AGG GTG GAC CAA TCA GGA GA-3′
		Reverse	5'-CCG CAT CAG TGA AAG TGT GC-3′
NM_010024.3	*Trp2 (mouse)*	Forward	5'-CCT GAA TGG GAC CAA TGC CT-3′
		Reverse	5'-GAA AAG CCA GCA ACC CCA AG-3′
NM_201394.2	*Plectin (mouse)*	Forward	5'-GCT TTC GAT CAC TGA GTT TGC-3′
		Reverse	5'-ACT GAT GGG GTA GGA GGA AGA-3′
NM_001276764.1	*Bp230 (mouse)*	Forward	5'-CAC CGC GAG CAG ATA ATA GAG-3′
		Reverse	5'-GAG CAG GTT CTT GAT GAG CAC-3′
NM_009842.3	*Cd151 (mouse)*	Forward	5'-CAT TAG TCT GCT GGC CTC AAG-3′
		Reverse	5'-GTC ACC ATG ACA ACA ACA CCA-3′

Table S3. List of antibodies for Western blot (WB), Enzyme-linked immunosorbent assay (ELISA), and Immunohistochemistry (IHC).

Antibody	Company	Catalog No.	Dilution Rate		
			WB	ELISA	IHC
HO-1	Abcam	ab13248	1:500		
β-actin	Cell Signaling	4967	1:1,000		
8-OHdG	GeneTex	GTX41980		1:500	
NLRP3	BosterBio	M00034	1:500		
TXNIP	Cell Signaling	14715	1:1,000		
ASC	Santa Cruz	sc-271054	1:500		
Cleaved-caspase 1	Cell Signaling	89332	1:1,000		
Pro-caspase 1	Santa Cruz	sc-392736	1:500		
IL-18	Invitrogen	PA5-79481		1:500	
Laminin	Novus Biological	NB300-144			1:200
Nidogen	Santa Cruz	sc-47773			1:50
Collagen IV	Invitrogen	PA1-28534			1:100
p38	Cell Signaling	9212	1:1,000		
pp38	Cell Signaling	4511	1:1,000		
PKA	Santa Cruz	sc-390548	1:500		
CREB	Sigma-Aldrich	C8977	1:1,000		
pCREB	Cell Signaling	9198	1:1,000		
MITF	LSBIO	LS-C117668	1:500		
pMITF	BT LAB	BT-AP11347	1:500		
